# Direct single-molecule visualization of Hsp90-mediated relief of an Hsp70-folding block

**DOI:** 10.1126/sciadv.aeg5464

**Published:** 2026-07-29

**Authors:** Nicholas R. Marzano, Bailey Skewes, Shannon McMahon, Lauren Rice, Dezerae Cox, Antoine M. van Oijen, Heath Ecroyd

**Affiliations:** ^1^ Molecular Horizons and School of Science, University of Wollongong, Wollongong, NSW 2522, Australia.; ^2^ Yusuf Hamied Department of Chemistry, University of Cambridge, Cambridge CB2 1EW, UK.; ^3^ UK Dementia Research Institute, University of Cambridge, Cambridge CB2 0AH, UK.; ^4^ Faculty of Medicine and Health, University of Sydney, G02 Jane Foss Russell Building, Sydney, NSW 2006, Australia.

## Abstract

Heat shock protein 70 (Hsp70) and Hsp90 are essential molecular chaperones that cooperate to fold diverse client proteins, yet how their activities are coordinated to remodel clients remains unclear. To address this, we used a combination of single-molecule fluorescence resonance energy transfer and total internal reflection fluorescence microscopy to observe individual firefly luciferase proteins during sequential engagement with *Escherichia coli* Hsp70 (DnaK) and Hsp90 (HtpG). We show that HtpG reduces rebinding of DnaK to folding intermediates while still allowing engagement with misfolded clients, enabling productive refolding in the presence of typically inhibitory concentrations of DnaK. HtpG couples adenosine 5′-triphosphate binding and hydrolysis to promote progressive folding through localized compaction across multiple regions of the client, reducing misfolding and establishing native interdomain contacts. Kinetic simulations support a model whereby heterogeneous DnaK binding generates region-specific folding kinetics and conformational dynamics. This enables efficient subdomain folding by DnaK/HtpG and suggests that the number and position of DnaK binding sites on clients provide a mechanism by which proteins can harness chaperone promiscuity for optimal folding.

## Teaser

Hsp90 promotes protein folding by reducing Hsp70 rebinding and enabling progressive client remodeling.

## INTRODUCTION

Most proteins need to fold into specific, three-dimensional (3D; native) conformations to perform their biological functions (*1*). In the cell, however, folding represents a challenging problem for a protein due to the large number of conformations that a polypeptide can adopt (*2*) and their propensity to enter “off-folding” pathways, which can lead to the accumulation of non-native, misfolded structures. These can result in diseases associated with protein loss-of-function (e.g., cystic fibrosis) (*3*) or protein aggregation, including numerous neurodegenerative disorders (e.g., Alzheimer’s and Parkinson’s diseases) (*4*, *5*). Consequently, cells have evolved a vast and diverse network of molecular chaperones to assist in the de novo folding and refolding of misfolded proteins to their native conformations (*6*). Among the most studied molecular chaperones are the intracellular heat shock proteins (Hsps), notably the Hsp70 and Hsp90 systems, which work cooperatively to guide polypeptides toward their native state (*7*, *8*).

The current model by which Hsp70 and Hsp90 cooperate to fold proteins is best established for the *Escherichia coli* system, whereby a Hsp40 cochaperone (DnaJ) binds to a client protein and delivers it to adenosine 5′-triphosphate (ATP)–bound Hsp70 (DnaK). DnaJ stimulates ATP hydrolysis 1000-fold (*9*), which causes large-scale conformational rearrangements in DnaK that result in ultrastable capture of the client protein by Hsp70. Binding of multiple Hsp70 proteins to a single client induces substantial client expansion to resolve misfolded states (*10*, *11*). Association of a nucleotide exchange factor (NEF; GrpE in *E. coli*) accelerates exchange of adenosine 5′-diphosphate (ADP) for ATP on Hsp70, which promotes release of the client and provides an opportunity for productive folding; multiple cycles of client binding and release eventually drive folding to the native state (*11*). However, recent evidence suggests that high, yet physiologically relevant, concentrations of Hsp70 promote excessive binding of the client protein by Hsp70, which effectively blocks refolding; when this occurs, transfer of the client to Hsp90 (HtpG) alleviates Hsp70-induced inhibition and allows folding to proceed (*12*), even at substoichiometric levels. It is well established that Hsp90 function is driven by large-scale conformational rearrangements associated with ATP binding and/or hydrolysis, with many structural and single-molecule analyses describing these conformational changes in Hsp90 (*13*–*15*). However, there are a number of outstanding questions that remain with regard to the client protein during cooperative folding by Hsp70 and Hsp90 including (i) how do the nucleotide-regulated changes in the chaperones influence the conformation of the client protein so as to promote protein folding?; (ii) how does Hsp90 protect the client from Hsp70-mediated inactivation?; and (iii) what is the role of ATP binding versus hydrolysis on the conformation of the client protein? In our previous single-molecule study (*11*), we showed that DnaK binding conformationally expands firefly luciferase (Fluc) to resolve misfolded states and that repeated binding and release cycles promote refolding. However, how these DnaK-driven conformational changes are coupled to productive folding by Hsp90 remains unclear. Furthermore, it is not well understood how heterogeneous chaperone interactions enables multidomain proteins to fold productively while avoiding misfolding.

A difficulty in addressing these outstanding questions is the heterogeneous, dynamic and transient nature by which these chaperones interact with client proteins, which substantially complicates efforts to identify and characterize important processes. While nuclear magnetic resonance (*16*–*18*) and structural studies (*19*–*21*) have been used to visualize the conformation of clients bound to Hsp90, these only provide static representations of proteins trapped in defined states (e.g., ATP versus ADP bound). Hence, real-time observation of client conformational dynamics throughout the entire Hsp70/Hsp90 cycle is not possible from protein structures alone, and the contribution(s) of these dynamics to protein folding are not well understood. To overcome this limitation, we performed single-molecule fluorescence resonance energy transfer (smFRET) experiments to temporally monitor the conformation of individual molecules of the multidomain client protein Fluc, as it is sequentially engaged and folded by the bacterial (*E. coli*) Hsp70 and Hsp90 systems.

We find that HtpG uses ATP hydrolysis to facilitate client escape from DnaK-mediated folding inhibition by reducing DnaK client binding and promoting progressive, stepwise folding events that enhance the probability of correct folding and reduce misfolding. These mechanisms are conserved across different domains of Fluc, suggesting a general mechanism by which HtpG uses ATP hydrolysis to fold clients. smFRET and kinetic simulations indicate that DnaK binds dynamically to multiple sites on Fluc, with regional differences in the number of DnaK binding sites controlling client expansion kinetics; this enables DnaK and HtpG to spatially separate domains, allowing subdomains to fold independently and efficiently. Collectively, we show that Hsp70/Hsp90-mediated folding provides a mechanism to rescue kinetically trapped proteins and subsequently smooth the folding landscape, thereby enabling rapid client folding with high fidelity.

## RESULTS

### HtpG blocks DnaK binding and promotes progressive folding

**Fig. 1.**
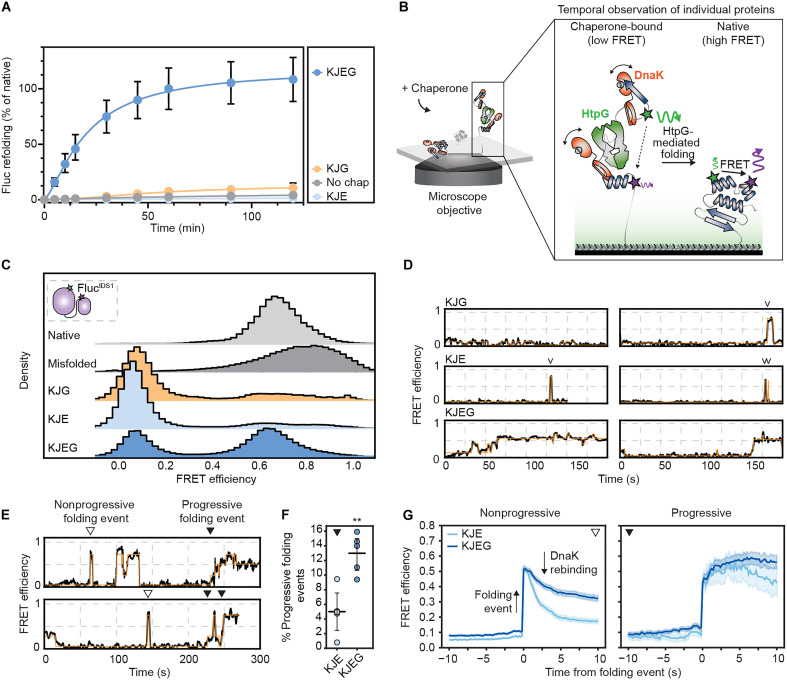
HtpG prevents the DnaK-mediated inhibition of client refolding by promoting progressive folding events. (**A**) Luciferase refolding assay in the absence or presence of the KJEG system. Fluc^IDS1^ (10 nM final concentration) was diluted 100-fold into refolding buffer alone (i.e., no chap) or supplemented with various combinations of molecular chaperones. Data shown represent the mean ± SEM from three independent experiments. (**B**) Schematic of the smFRET imaging setup. Alexa Fluor 555 (AF555)/Alexa Fluor 647 (AF647)–labeled Fluc^IDS1^ was immobilized to a neutravidin-functionalized coverslip and was illuminated by a 532-nm laser that selectively excites the AF555 donor fluorophore. The fluorescence from both AF555 and AF647 (acceptor) fluorophores was measured over time in the absence or presence of chaperones, and the FRET efficiency was calculated. (**C**) FRET efficiency histograms of Fluc^IDS1^ (inset; relative position of FRET pair is denoted) in the native state, misfolded or during 60 min of incubation with the indicated combination of chaperones. Data are compiled from at least 193 individual trajectories for each condition from three independent experiments. (**D**) Example FRET trajectories from individual Fluc^IDS1^ molecules upon incubation with the indicated combination of molecular chaperones [hidden Markov model (HMM) shown in orange]. Arrows indicate the presence of spike events. (**E**) Example FRET trajectories showing folding events away from the DnaK-bound state (i.e., transitions from <0.3 to >0.3 FRET) classified into either nonprogressive (empty triangles) or progressive events (filled triangles). (**F**) The percentage of folding events classified as progressive. Data are presented as mean ± SEM from at least five independent repeats. **P < 0.01 following an unpaired *t* test. (**G**) The average FRET efficiency before and after nonprogressive (left) and progressive (right) folding events ± SEM. Data are compiled from at least 1242 events per condition.

First, we established that the bacterial Hsp90 system (consisting of DnaK, DnaJ, GrpE, and HtpG, denoted as KJEG) could refold a denatured client protein. For these experiments, we used a previously reported Fluc mutant containing introduced cysteines on each Fluc domain (denoted as Fluc^IDS1^ because this protein can act as an interdomain sensor of protein folding via smFRET) (*11*). Fluc^IDS1^ refolding was monitored by measuring the luminescence from correctly folded and native protein over time. The complete KJEG system was required to refold Fluc^IDS1^ to an enzymatically active state (Fig. 1A) and was dependent on GrpE-mediated handover of the client to HtpG because omission of either GrpE or HtpG prevented refolding. Notably, the inability of the KJE system to refold Fluc^IDS1^ in the absence of HtpG is due to the high (yet physiologically relevant) concentrations of DnaK used in this assay (*11*, *12*).

Having established that the KJEG system can refold Fluc^IDS1^ to an enzymatically active state, we next investigated how the conformation of individual Fluc^IDS1^ proteins is altered during HtpG-mediated refolding using a combination of total internal reflection fluorescence (TIRF) microscopy and smFRET (Fig. 1B). Native Fluc^IDS1^ exhibited a single FRET distribution centered at ∼0.6, which increased to ∼0.8 upon formation of a chemically induced misfolded state (Fig. 1C), consistent with our previous work (*11*). Incubation of misfolded Fluc^IDS1^ with DnaJ/DnaK in the absence of either GrpE or HtpG, which was previously shown to inhibit refolding (Fig. 1A), resulted in the formation of an ultralow FRET state (centered at ∼0.1). We (and others) have previously shown that this state is characteristic of DnaK-bound Fluc^IDS1^, whereby binding of many DnaK proteins results in substantial conformational expansion of the client to resolve misfolded states (*10*, *11*). Analysis of individual Fluc^IDS1^ molecules from these conditions revealed that the DnaK-bound state was extremely long lived but would occasionally exhibit rapid FRET efficiency spikes, indicative of DnaK dissociation (i.e., an increase in FRET) followed by rapid rebinding (i.e., a decrease in FRET) (Fig. 1D and fig. S1, A and B). Because escape from DnaK-bound states is accelerated by the NEF GrpE (*11*), such folding events are generally attributed to stochastic DnaK dissociation events. These data are consistent with Fluc^IDS1^ becoming trapped within the DnaK-bound state, which is not permissible for productive refolding. Refolding of Fluc^IDS1^ was only observed in the presence of the complete KJEG system, with transitions away from the ultralow FRET (DnaK-bound) state toward a native-like FRET efficiency often occurring gradually or in rapidly sequential steps, consistent with progressive folding of the client. These progressive folding events, during which hydrophobic regions are potentially shielded to form the hydrophobic core of the protein, may explain how HtpG can assist Fluc^IDS1^ to escape rebinding by DnaK.

Because progressive increases in FRET from the DnaK-bound state appeared to be more frequent in the presence of HtpG, we classified transitions away from the DnaK-bound state (i.e., when FRET increased from <0.3 to >0.3) as being either those in which the next change in FRET was an increase (a classification we defined as progressive) or those in which the FRET did not change or the next change was a decrease (a classification we defined as nonprogressive; Fig. 1E). There was a ∼2.5-fold higher probability of progressive folding events when HtpG was present compared to when HtpG was omitted (Fig. 1F). Plotting the average FRET efficiency before and after these folding events (Fig. 1G) shows that, for nonprogressive events, the FRET efficiency decreased rapidly in the absence of HtpG (indicative of Fluc^IDS1^ misfolding and subsequent rebinding by DnaK); however, the rate and amount of DnaK rebinding were substantially less in the presence of HtpG (Fig. 1G). This could be due to either HtpG-mediated refolding being more likely to result in the client adopting a native-like fold, HtpG preventing DnaK rebinding, or a mixture of both. Fitting the DnaK rebinding curves to a single exponential supports it being a mixture of both mechanisms (fig. S1, C and D) because HtpG reduces both the rate constant (indicating slower DnaK rebinding rates) and the total amplitude (indicating reduced DnaK engagement on Fluc^IDS1^ due to native folding or HtpG-mediated blocking). To interrogate this further, we plotted the same data as 2D FRET histograms to visualize the true FRET efficiencies following nonprogressive folding events. As expected, most folding events result in Fluc^IDS1^ becoming rapidly reengaged by DnaK and returned to the low-FRET state (fig. S2), while a smaller subset of molecules remain at native-like FRET efficiencies, indicative of successful refolding and consistent with our analysis of the averaged data (Fig. 1G).

Conversely, progressive folding events were characterized by gradual or stepwise increases in FRET efficiency for all conditions tested (Fig. 1G). Crucially, no reduction in FRET efficiency was observed after the folding event (indicative of minimal DnaK rebinding). This suggests that progressive folding events, which are promoted by HtpG (Fig. 1F), are more likely to result in Fluc^IDS1^ reaching a native fold compared to nonprogressive events. This is especially evident in the 2D histograms, in which Fluc^IDS1^ often passes through a folding intermediate of moderate FRET efficiency (∼0.4) before transitioning to either (i) a high-FRET misfolded state, (ii) a native-like FRET state, or (iii) a low-FRET DnaK-bound state (fig. S2). Because of partial overlap in FRET efficiencies of native and misfolded states (∼0.6 to 0.8), we consider both FRET and DnaK binding behavior when interpreting folding outcomes, with the absence of DnaK rebinding (i.e., no return to the low-FRET expanded state) providing the most reliable indicator of a native-like conformation. Consistent with the analysis of the averaged data, HtpG-mediated progressive refolding resulted in more stable native-like FRET efficiencies and less misfolded or DnaK-bound states.

### ATP hydrolysis by HtpG coordinates folding with DnaK and client

**Fig. 2.**
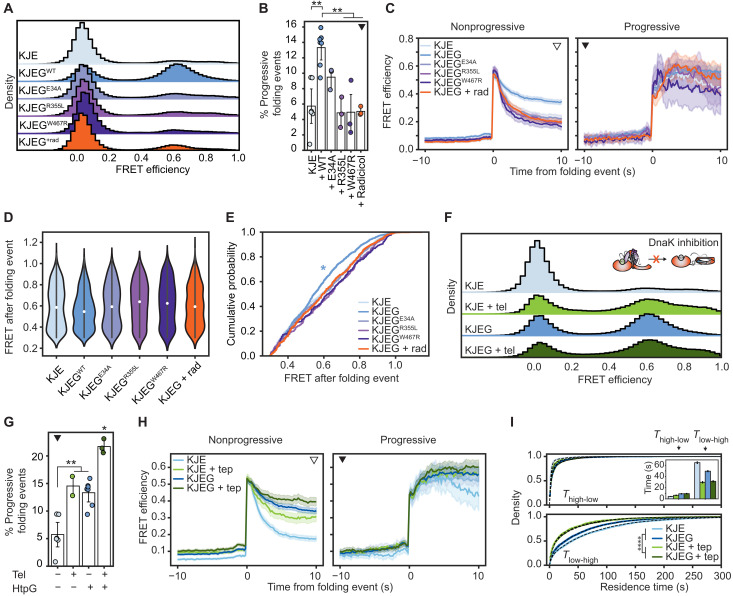
HtpG selectively prevents DnaK rebinding during folding events. (**A**) FRET efficiency histograms of Fluc^IDS1^ during 60 min of incubation with the indicated combination of chaperones/inhibitors (60 μM radicicol). Data are compiled from at least 686 individual trajectories for each condition. Rad, radicicol. (**B**) The percentage of folding events that are classified as progressive. (**C**) The average FRET efficiency before and after nonprogressive (left) and progressive (right) folding events. Data are compiled from at least 431 events per condition from three independent experiments. (**D**) The distribution of Fluc^IDS1^ FRET states immediately following transitions from the DnaK-bound state used for distribution analysis in panel (E). (**E**) Cumulative histogram of the FRET state immediately following transition from the DnaK-bound state. Data were analyzed using a Kolmogorov-Smirnov test with bootstrapping for differences in the distribution. * denotes statistically significant difference (*P* < 0.05) between KJEG and all other conditions. (**F**) FRET efficiency histograms of Fluc^IDS1^ during 60 min of incubation with the indicated combination of chaperones/inhibitors (100 μM telaprevir). Data are compiled from at least 1207 individual trajectories for each condition from three independent experiments. Tel, telaprevir. (**G**) The percentage of folding events that are classified as progressive (mean ± SEM). (**H**) The average FRET efficiency before and after nonprogressive (left) and progressive (right) folding events ± SEM. Data are compiled from at least 432 events per condition. (**I**) Cumulative residence time data showing the time Fluc^IDS1^ remains in a DnaK-bound state (i.e., <0.3 FRET) before a nonbound state (i.e., *T*_low-high_) and vice versa. The average residence time (inset) is calculated and presented as mean ± SEM for clarity. For panels (B), (G) and (I), a one-way analysis of variance (ANOVA) with Tukey’s post hoc test was performed, with *P < 0.05, **P < 0.01, and ****P < 0.001.

To interrogate how Hsp90 uses ATP and cooperates with the Hsp70 system to promote efficient client refolding, we performed smFRET experiments in the presence of an HtpG inhibitor (radicicol, which prevents ATP binding) or with an ATPase–deficient HtpG mutant (HtpG^E34A^). Both conditions could not refold Fluc^IDS1^ (Fig. 2A and fig. S3, A and example traces in B) and exhibited a reduced capacity to promote progressive folding events compared to wild-type HtpG (Fig. 2B). Crucially, control experiments performed with HtpG under steady-state ATP conditions (via an ATP regeneration system) yielded identical folding behaviors (fig. S4), indicating that the observed results are not substantially affected by ADP accumulation. HtpG^E34A^ also promoted higher rates of progressive folding events compared to radicicol-treated HtpG, indicating that ATP binding alone (or even slow rates of ATP hydrolysis) is important in facilitating progressive folding events (Fig. 2B). We next tested previously described HtpG mutants that are defective in their interactions with DnaK (HtpG^R355L^) or the client (HtpG^W467R^) (*8*). Both mutants also reduced the occurrence of progressive folding events and failed to support Fluc^IDS1^ refolding (Fig. 2, A and B, and fig. S3, A and B), indicating that productive transitions require HtpG to interact with both DnaK and unfolded Fluc^IDS1^. As before, progressive folding events were characterized by minimal DnaK rebinding (indicative of correct folding; Fig. 2C), while only the functional wild-type HtpG system could reduce DnaK reengagement and improve folding outcomes following nonprogressive events. Together, these data support a model in which progressive transitions arise within a DnaK-HtpG-client ternary complex that promotes productive folding from the DnaK-bound state.

We further investigated how HtpG promotes optimal client refolding by looking at the first FRET state of an individual Fluc^IDS1^ molecule immediately following escape from the DnaK-bound state [Fig. 2D; based on hidden Markov model (HMM) fits]. A distribution analysis revealed that HtpG significantly reduces transitions to typically misfolded high-FRET Fluc^IDS1^ structures (i.e., >0.6) compared to when ATP binding and/or hydrolysis is inhibited (i.e., with HtpG^E34A^ or radicicol; Fig. 2E) or its interaction with either DnaK (HtpG^R355L^) or the client (HtpG^W467R^) is impaired. This finding is consistent with previous experiments that suggest that ATP hydrolysis by HtpG is critical in controlling client refolding (*22*). Wild-type HtpG also accelerates escape from DnaK-bound states [observed as a decrease in *T*_low-high_ (see Methods); fig. S3C] compared to the HtpG mutants tested, which allows productive folding events to occur more often. Collectively, these data suggest that, through direct interactions with DnaK and Fluc^IDS1^, HtpG uses ATP hydrolysis to increase the probability of Fluc^IDS1^ folding toward the native state by actively protecting it from forming nonproductive misfolded intermediates following escape from DnaK-bound states.

### HtpG selectively protects folding intermediates

Because our data suggest that HtpG prevents DnaK rebinding during folding, we investigated whether optimal refolding could be recapitulated by reducing the binding activity of DnaK. Addition of the DnaK-specific inhibitor telaprevir to typically inhibitory concentrations of DnaK (Fig. 1A) allowed Fluc^IDS1^ refolding to be restored (albeit at a slower rate) in the absence of HtpG and completely restored in the presence of HtpG (fig. S5A). This suggests that (i) reducing DnaK-association rates improves folding and (ii) DnaK is not completely inhibited at these concentrations of telaprevir, consistent with previous work (*23*). Repeating these experiments at the single-molecule level yielded similar results, whereby the addition of telaprevir improved refolding yields in the absence of HtpG (Fig. 2F and example traces in fig. S5B), but not during HtpG-mediated folding. Telaprevir resulted in a substantial increase in misfolded Fluc^IDS1^ for both conditions (fig. S5C). Telaprevir-induced DnaK inhibition resulted in an increased propensity of progressive folding events relative to the noninhibited controls (i.e., with or without HtpG; Fig. 2G) and, as expected, reduced the rate of DnaK rebinding following attempted folding events (Fig. 2H and fig. S5, D and E). Notably, the increase in progressive folding events under both telaprevir- and HtpG-treated conditions indicates that these transitions report on similar underlying folding processes. However, the additive effect of HtpG in the presence of telaprevir suggests that comparable FRET signatures can arise from distinct mechanisms, including reduced DnaK rebinding and direct client remodeling by HtpG (likely mediated by ATP hydrolysis; see Fig. 2B). These observations suggest that (i) HtpG is more effective than telaprevir at slowing DnaK rebinding (fig. S5E) and (ii) HtpG selectively inhibits DnaK rebinding to Fluc^IDS1^ molecules undergoing progressive folding while still allowing DnaK to engage misfolded species. The latter point is evidenced by a lower abundance of misfolded species when HtpG is present (fig. S5C; because DnaK is still active and can bind misfolded protein), whereas telaprevir broadly inhibits DnaK binding to all Fluc^IDS1^ states. Collectively, these data indicate that HtpG selectively inhibits DnaK rebinding to HtpG-bound clients, enabling productive folding attempts without reducing the high association rates of DnaK with misfolded client proteins.

The addition of telaprevir also resulted in substantially faster escape from DnaK-bound states compared to HtpG-mediated folding alone (Fig. 2I). Because longer *T*_low-high_ residence times are correlated with greater DnaK occupancy on the client (*11*), our data suggest that fewer DnaK molecules engage Fluc^IDS1^ under these conditions than during HtpG-mediated folding. This reinforces earlier findings that HtpG does not interfere with the capacity of DnaK to bind to and protect misfolded client proteins (as reflected by longer *T*_low-high_ residence times) but rather permits progressive folding events that would not typically occur in its absence. Notably, the effect of telaprevir on KJE-mediated folding (i.e., improved refolding, faster escape from DnaK-bound states, and occurrence of progressive folding events) could be closely recapitulated by simply reducing the concentration of DnaK fourfold (fig. S4). Together, these data support a mechanism whereby slower DnaK association rates during folding events, mediated either by HtpG, telaprevir, or reduced DnaK concentrations, result in more progressive folding processes and improved Fluc^IDS1^ folding outcomes, potentially because multiple asynchronous DnaK dissociation events can occur during domain folding without interference from DnaK rebinding.

### HtpG drives progressive intra- and interdomain folding

**Fig. 3.**
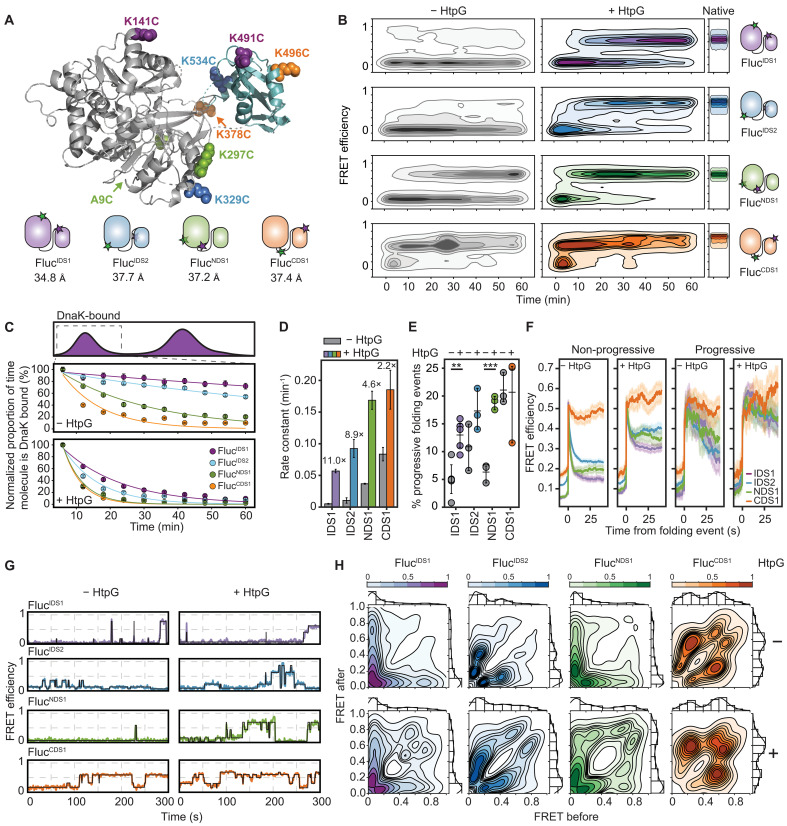
HtpG accelerates the folding of all Fluc domains and is critical for establishing native interdomain contacts. (**A**) Crystal structure of Fluc [ribbon; N-terminal domain (gray) and C-terminal domain (blue) are denoted; Protein Data Bank: 1LCI (*63*)] with the position of introduced cysteines for each smFRET sensor denoted (spheres). Cartoon representation of the approximate position and distance (in angstroms, as measured from the Cα of each residue) of each FRET pair on Fluc is shown below. We note that the actual position of the dyes may vary because they do not include estimations of the attached linker regions. (**B**) FRET intensity heatmaps for each Fluc sensor when incubated in the presence of the KJE system alone (−HtpG) or in the presence of HtpG (+HtpG). Native Fluc is shown on the right. Data are compiled from at least 1252 individual trajectories for each condition from three independent experiments. (**C**) The proportion of time that each Fluc protein is DnaK bound (i.e., <0.3 FRET), shown as the mean ± SEM of all molecules from each time point. Calculated from data presented in panel (B). Data for each condition were fit with an exponential decay curve. (**D**) The rate of refolding of each Fluc sensor derived from the rate constants calculated from the exponential fit in (C). The fold increase in refolding rate when HtpG is present is denoted. (**E**) The percentage of folding events classified as progressive when refolded by the KJE system alone or supplemented with HtpG. Each Fluc sensor in the absence or presence of HtpG was statistically analyzed by an unpaired *t* test, with **P < 0.05 and ***P < 0.01. Data come from at least three independent experiments. (**F**) The average FRET efficiency before and after nonprogressive (left) and progressive (right) folding events ± SEM. Data are compiled from at least 115 events per condition. (**G**) FRET trajectories for each Fluc sensor when incubated in the presence of the KJE system only or when supplemented with HtpG. (**H**) Transition density plots (TDPs) of each Fluc sensor when incubated in the presence of the KJE system only (top) or when supplemented with HtpG (bottom). The normalized occurrence of each transition is colored according to the density colorbars (above).

Because Fluc^IDS1^ reports only on the distance between the N- and C-terminal domains, we developed additional cysteine mutants that allow us to observe the folding of other Fluc regions. To this end, FRET pairs were positioned to generate another interdomain sensor that also reports on the domain linker (Fluc^K329C-K534C^; denoted as Fluc^IDS2^), an N-terminal domain sensor (Fluc^A12C-K329C^; denoted as Fluc^NDS1^), and a C-terminal domain sensor (Fluc^K378C-K496C^; denoted as Fluc^CDS1^) (Fig. 3A). Refolding of all Fluc constructs was performed in the absence or presence of HtpG. Both the FRET efficiency and proportion of time that each molecule spends in an ultralow (DnaK-bound) FRET state were determined and act as an inverse measure of the amount of native protein and thus refolding. In the absence of HtpG, both interdomain sensors (i.e., Fluc^IDS1^ and Fluc^IDS2^) remained predominantly DnaK bound over time (Fig. 3, B and C), while the intradomain sensors could partially (in the case of Fluc^NDS1^) or completely (for Fluc^CDS1^) escape the DnaK-trapped state and return to more native-like FRET efficiencies (Fig. 3B). However, in the presence of HtpG, the rate of refolding (determined by fitting the decay curve of DnaK-bound molecules; Fig. 3C) was ∼11-fold faster for Fluc^IDS1^, ∼9-fold for Fluc^IDS2^, ∼4.5-fold for Fluc^NDS1^, and ∼2-fold for Fluc^CDS1^ (Fig. 3D), with all sensors returning to native-like FRET efficiencies. HtpG did not alter the order in which each Fluc domain folded; from fastest to slowest, this was Fluc^CDS1^ > Fluc^NDS1^ > Fluc^IDS2^ > Fluc^IDS1^. After 15 min, the interdomain regions probed by Fluc^IDS1^ and Fluc^IDS2^ were DnaK bound (i.e., completely unfolded) ∼40 to 60% of the time compared to only 15% for the N- and C-terminal domain sensors. While it has previously been suggested that the Hsp70-Hsp90 cascade increases refolding yields but not folding kinetics (*12*), the much faster rates observed here in the presence of HtpG likely reflect the relatively poor refolding ability of AviTagged Fluc, which necessitates a higher dependence on chaperone function for folding (*11*). Regardless, these data suggest that (i) interdomain folding comes with more kinetic traps (and thus more critically necessitates the action of HtpG), (ii) the N- and C-terminal domains fold independently to prevent misfolding, (iii) the formation of native interdomain contacts is the final stage of folding and requires both N- and C-terminal domains to be mostly (if not completely) folded, and (iv) HtpG accelerates the rate of refolding but does not change the order in which each domain folds.

We next looked at whether HtpG also promoted progressive folding of other regions of Fluc. Because all Fluc sensors transition to an ultralow (<0.3) FRET state upon DnaK binding, we applied the same criteria for defining progressive or nonprogressive transitions as previously described for Fluc^IDS1^ to allow meaningful comparison of the folding of different Fluc regions. HtpG significantly increased the proportion of progressive folding events within the N-terminal domain (i.e., Fluc^NDS1^) and trended higher for Fluc^IDS2^ (Fig. 3E); notably, there was no effect within the C-terminal domain (i.e., Fluc^CDS1^), which is the region of Fluc that is least dependent on chaperone intervention for refolding (*10*).

Consistent with observations from Fluc^IDS1^, DnaK rebinding following nonprogressive folding (as indicated by a decrease in FRET efficiency) was substantially less for all Fluc sensors when in the presence of HtpG (Fig. 3F), except for Fluc^CDS1^. The FRET efficiency before nonprogressive folding events (i.e., those most likely to result in misfolding and DnaK rebinding) was slightly lower for Fluc^IDS1^ and Fluc^NDS1^ (∼0.05) in the absence of HtpG compared to when HtpG was present (∼0.08; Fig. 3F and fig. S6A), which although is a small change in FRET efficiency actually constitutes a substantial (∼1 nm) difference in distance due to the nonlinear (inverse sixth-power) relationship between donor-acceptor distance on FRET efficiency (fig. S6B). Notably, Fluc regions characterized by low FRET efficiencies before folding events were correlated with longer *T*_low-high_ residence times (fig. S6C) and a higher dependence on HtpG to facilitate progressive folding (Fig. 3E). Thus, these data suggest that binding of HtpG to Fluc reduces DnaK-mediated client expansion, shifting the population toward less-expanded conformations, although similar states can also be accessed during refolding in the absence of HtpG.

Moreover, progressive folding events resulted in less DnaK rebinding for all Fluc sensors, especially in the presence of HtpG where minimal rebinding was observed even 40 s after the initial folding attempt (Fig. 3F). Notably, 2D FRET histograms of both progressive and nonprogressive folding events showed that Fluc^IDS2^ and Fluc^NDS1^ transiently populate intermediate FRET states (∼0.4 to 0.5) en route to acquiring the native state, which is even more pronounced when HtpG is present (fig. S7). Although the intermediate FRET state appears to be a general feature of Fluc folding, transitions to higher-FRET native conformations are selectively enhanced and stabilized by HtpG. Collectively, these data are consistent with HtpG promoting progressive folding events through distinct folding intermediates that result in more native-like conformations.

By filtering the data for those molecules that are DnaK bound [i.e., visit FRET states < 0.3, which is a prerequisite for HtpG function (*24*)], we interrogated how HtpG regulates transitions between different Fluc conformational states during productive folding. Individual FRET traces were fit with an HMM (Fig. 3G and additional traces in fig. S6D), and the distribution of FRET transitions is represented as a function of the FRET state before and after each transition as a transition density plot (TDP; Fig. 3H). In the absence of HtpG, Fluc molecules were often characterized by short-lived spikes from an ultralow-FRET DnaK-bound state to either intermediate states (∼0.4 for Fluc^IDS2^) or high-FRET misfolded states (for Fluc^IDS1^ and Fluc^NDS1^); this is observed at the population level by high transition density along the TDP axes. Fluc^IDS2^ appeared to report on exchanges between two different DnaK-bound states (with FRET efficiencies of ∼0.1 and 0.4, respectively). Because such low FRET states are not present in the absence of DnaK, these may be due to local folding of the linker and/or C-terminal domain upon DnaK association and dissociation. This is supported by residence time data, whereby transitions away from the ultralow-FRET DnaK-bound state occur at similar time frames for Fluc^CDS1^ and Fluc^IDS2^ (fig. S6C). Collectively, however, all Fluc sensors are constrained within DnaK-bound states in the absence of HtpG (except for Fluc^CDS1^), whereby escape from DnaK-bound states is more often followed by DnaK rebinding rather than productive folding events. In addition, we observed strong transition density within the low-FRET DnaK-bound population (i.e., ∼0.05 to 0.25 FRET) for all Fluc variants except Fluc^CDS1^. These data suggest that, for the variants displaying this behavior, the chaperone-bound ensemble includes multiple DnaK-bound states due to binding at different sites or in varying numbers (Fig. 3H).

Crucially, HtpG alleviated the DnaK-induced block for all Fluc sensors by promoting transitions from intermediate FRET states (∼0.2 to 0.5) to more native-like conformations (∼0.6 to 0.8), especially for Fluc^IDS2^ and Fluc^NDS1^ (Fig. 3G), consistent with a higher proportion of progressive folding events (Fig. 3F). At the population level, these are evident as increased transition density along the top axis of the TDPs (Fig. 3H) and are also evident in the 2D FRET heatmaps (figs. S2 and S7). Fluc^IDS2^ often dynamically transitioned between an intermediate FRET state (∼0.6) and the native state (∼0.8) (fig. S6D), with transitions to the native state increasing over time (fig. S6E). In addition, transitions away from the native state were poorly tolerated at early time points (i.e., the FRET efficiency remained low and did not return to native-like states), while those at later stages of folding recovered faster (i.e., quickly returned to the native state; fig. S6F). Collectively, the transition of Fluc^IDS2^ between two distinct states (likely due to DnaK association and dissociation at a single binding site) suggests that the folding of other protein regions (e.g., Fluc^NDS1^ or Fluc^CDS1^) is a prerequisite for stable Fluc^IDS2^ folding.

While there is a preference for stepwise domain folding [i.e., C-terminal repeat domain (CTD) > N-terminal domain (NTD) > interdomain], DnaK dissociation is an intrinsically stochastic process. Hence, it is likely that the conformational fluctuations observed within each Fluc sensor occur simultaneously within individual proteins and that folding of different domains or regions occurs, while other regions are spatially separated to prevent aberrant misfolding events. A key question arising from this is how a stochastic process (DnaK dissociation) can drive subdomain folding in a relatively conserved order. This work suggests that the inherent properties of the client dictates ordered folding, whereby (i) certain (sub)domains fold more efficiently than others (e.g., as observed for the C-terminal domain of Fluc) and/or (ii) the order of subdomain folding is regulated by the propensity or occurrence of molecular chaperone binding (e.g., regions with more DnaK binding sites fold slower). We sought to further explore this latter hypothesis by investigating how long each Fluc variant spends in the DnaK-bound state before folding events.

### Heterogeneous DnaK binding drives region-specific folding

When we interrogated the distribution of DnaK-dissociation events (i.e., *T*_low-high_; fig. S6C), the data were best described by a double exponential with two rate constants (fig. S8, A and B); in general, this featured a relatively conserved fast rate (*k*_1_; ∼0.1 to 0.25 s^−1^) and a slow rate (*k*_2_; ∼0.01 to 0.03 s^−1^) (fig. S8C) for each Fluc sensor. For domain sensors with longer average residence times (i.e., Fluc^IDS1^ and Fluc^NDS1^), the slow rate was ∼2- to 3-fold slower compared to those sensors with shorter residence times (i.e., Fluc^CDS1^ and Fluc^IDS2^) and accounted for a larger portion of the overall fit (>70% versus 20 to 50%, respectively) (fig. S8C), indicating that this slow rate dominates the occurrence of Fluc refolding events for these sensors. Because DnaK dissociation is typically defined by a single exponential rate (*25*, *26*), we next explored why this simple on/off system was best described by a double exponential rate and why different regions of Fluc exhibited different residence times under the same conditions.

**Fig. 4.**
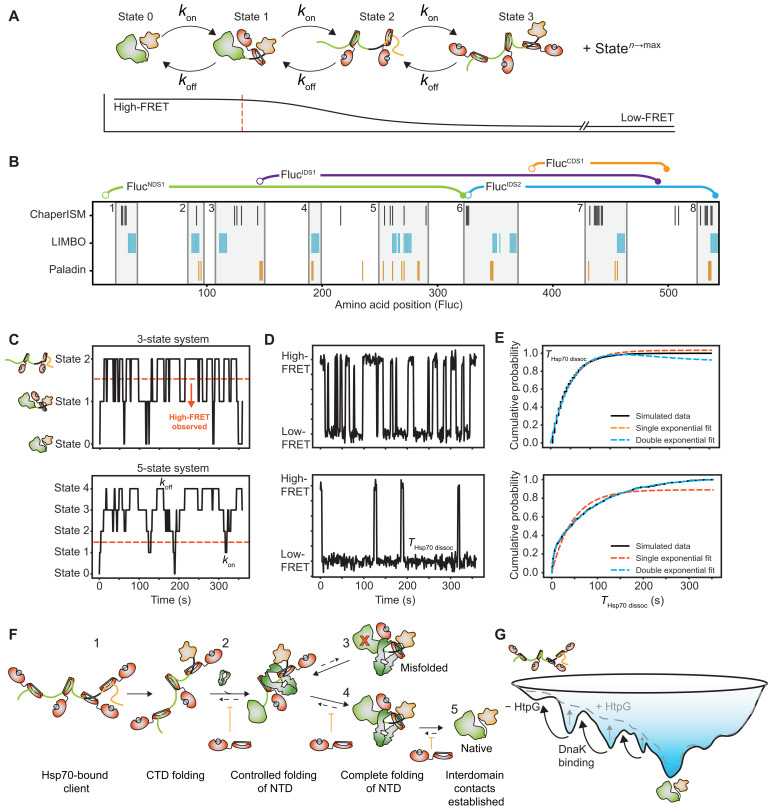
Dynamic exchange of DnaK between heterogeneous bound states regulates the kinetics of Fluc conformational expansion and folding. (**A**) Schematic illustrating the theoretical relationship between the number of client-bound DnaK molecules and the observed FRET efficiency of Fluc sensors. Because entropic pulling stipulates that binding of multiple Hsp70 subunits is required for full conformational expansion (i.e., low-FRET) (*62*), a threshold of <2 bound-DnaK proteins to observe high-FRET states was defined (orange line). Transitions between different DnaK-bound states are defined by a single experimentally derived *k*_on_ and *k*_off_. (**B**) Predicted DnaK binding sites on Fluc based on three different prediction algorithms (*29*–*31*). Regions of conserved DnaK binding between the prediction algorithms are highlighted and numbered (gray). Schematics showing the position of donor/acceptor dyes for each sensor (denoted as open/closed circles) are shown. The number of potential DnaK binding regions (gray regions) within Fluc^IDS1^ (5–6), Fluc^NDS1^ (5–6), Fluc^IDS2^ (3–4), and Fluc^CDS1^ (2–3) was determined, with binding regions within ∼40 amino acids of a fluorophore [which is the amino acid distance at which changes in FRET can be observed upon DnaK binding (*64*)] included in the count for that sensor. (**C**) Example simulated data, showing transitions between either three (top) or five (bottom) different possible DnaK-bound states. (**D**) FRET trajectories calculated from simulations in panel (B), where transitions to the “high-FRET” state only occur upon transitions to state 1 or lower. (**E**) The distribution of residence times from simulated FRET trajectories (C) was determined and plotted as cumulative density plots. Fits of the data to single- or double-exponential distributions are shown. (**F**) Model of HtpG-mediated folding of Fluc. (1) DnaK-mediated conformational expansion of Fluc occurs and is followed by (2) folding of the C-terminal domain in an HtpG-independent manner. The folding of the N-terminal domain occurs next in an HtpG-mediated process, whereby (3) HtpG uses ATP hydrolysis to promote progressive folding events, prevent DnaK rebinding, and minimize misfolding, which is assisted by spatial separation of N- and C-terminal domains through the HtpG lumen. Should misfolding occur at any stage (red X), (4) DnaK rebinding can still occur to resolve the misfolded state. (5) Upon independent folding of the N- and C-terminal domain, interdomain contacts are established in the final native state. (**G**) Mechanism of HtpG/DnaK-mediated folding. DnaK prevents the accumulation of misfolded client proteins in kinetic traps by returning them to a high entropy (i.e., conformationally expanded) state, while HtpG smooths the folding landscape to the native state.

One hypothesis is that the difference in observed residence times for different Fluc domains reflects the number of DnaK binding regions within that domain. On the basis of our smFRET data, we stipulate that the low-FRET (<0.3) state reflects a dynamic and heterogeneous ensemble of multiple DnaK molecules bound to a single Fluc client, consistent with the entropic pulling model of Hsp70 function in which simultaneous binding of several Hsp70 proteins help resolve misfolded states (Fig. 4A) (*11*, *27*). Analytical ultracentrifugation and fluorescence cross-correlation spectroscopy measurements suggest that up to ∼12 DnaK molecules can be present within Fluc-DnaK complexes; however, the number directly bound to the client is likely lower, based on peptide mapping studies and the ability of DnaK to engage its own interdomain linker (*10*, *28*). Escape from this expanded low-FRET conformation likely occurs upon dissociation of at least one DnaK, leaving one or no DnaK subunits bound, which results in a more compact conformation (FRET > 0.3). This assumption is supported by previous work (*11*), which demonstrated that low concentrations of DnaK result in partial binding and conformational expansion of Fluc to intermediate (∼0.3 to 0.6) FRET states. We then performed kinetic simulations of DnaK binding to a single Fluc molecule, using a fixed association (*k*_on_) and dissociation rate (*k*_off_; as determined from smFRET experiments; see Methods) to model the dynamic exchange between increasing numbers of DnaK-bound states. On the basis of predicted DnaK binding sites on Fluc from multiple prediction algorithms (*29*–*31*) and the position of fluorophores for each sensor, we assumed a maximum of two to five potential binding regions per Fluc sub(domain) and tracked the number of bound DnaK molecules over time (Fig. 4B). As expected, Fluc dynamically transitioned between different DnaK-bound states (Fig. 4C), spending a greater proportion of time bound by more DnaK molecules as the number of potential binding regions increased (fig. S8D).

To see whether this model reflects our experimental data, we assigned FRET values based on DnaK occupancy: low FRET for two or more bound DnaK proteins and high FRET when one or none were bound (Fig. 4D). This simplistic approach allowed us to test whether differences in experimentally observed residence times could arise from a heterogeneous ensemble of multiple bound states. When only three DnaK-bound states were possible, the simulated residence times were short (∼6 s) and fit well to a single exponential. Conversely, increasing the number of possible DnaK-bound states resulted in an increased average residence time with a distribution best described by a double exponential (consistent with our experimental smFRET data) (Fig. 4E). The simulated FRET trajectories also appeared similar to the experimental smFRET traces (Fig. 4C) and produced biexponential rate constants comparable to experimentally determined rates for our Fluc sensors (*k*_1_ ∼ 0.1 to 0.33 s^−1^; *k*_2_ ∼ 0.008 to 0.046 s^−1^; fig. S8E). While not encompassed in this simplistic model, it is possible that the intermediate FRET states observed experimentally during DnaK/HtpG-mediated folding (∼0.4 to 0.5; figs. S2 and S7) represent Fluc conformations while bound to a single DnaK protein. When the observed rate of DnaK rebinding to Fluc^IDS1^ following folding events in the presence of HtpG was used to define *k*_on_ in the simulations, only a minor decrease in residence times was observed (fig. S8F), consistent with experimental results (fig. S6C). Instead, the number of states was the most substantial determinant of residence times, which is consistent with the observation that Fluc sensors that spend longer in DnaK-bound states (i.e., Fluc^IDS1^ and Fluc^NDS1^; fig. S6C) contain more DnaK-binding regions than others (Fig. 4B). Hence, these data suggest that the number of potential DnaK binding regions plays a critical role in regulating Fluc refolding and explains how different Fluc domains exhibit different residence times under the same experimental conditions. This potentially provides a mechanism by which the generic chaperone function of Hsp70 and/or Hsp90 (i.e., client binding and release) can be modulated to accommodate the specific folding pathway of the client.

## DISCUSSION

We set out to describe the conformation of an individual client protein as it is engaged by the bacterial Hsp70/Hsp90 chaperone systems during productive folding using smFRET. In doing so, we found that HtpG (i) reduces DnaK rebinding during folding events, thereby allowing the client to escape from DnaK-induced inhibition, (ii) reduces client misfolding following the initial folding attempt, and (iii) promotes progressive folding of the client to the native state (Fig. 4F). Collectively, HtpG acts to release the Hsp70-induced folding block when levels of DnaK prevent productive folding and smooth the folding landscape to increase the rate of client folding to the native state (Fig. 4G).

HtpG increases productive folding of clients through enhancing the stability of folding intermediates and protecting against kinetic traps (as demonstrated by increased progressive folding events and reduced misfolding following DnaK release), with only subtle contributions from changes in chaperone-induced conformational kinetics (e.g., escape from DnaK-bound states and DnaK binding times). The improved folding stability is likely caused by a combination of factors. First, HtpG/DnaK-mediated chaperone folding spatially separates the N- and C-terminal domains, allowing each Fluc domain to refold independently (*32*) and preventing the erroneous interdomain contacts that can lead to misfolding. This interdomain misfolding is a common phenomenon and often a major folding bottleneck for many multidomain proteins (*33*, *34*). Our smFRET and simulation data suggest that the number and position of Hsp70 binding sites facilitate efficient separation of domains to prevent misfolding (via additional sites at interdomain regions) while still enabling intradomain folding events (via reduced binding sites). This effect is likely amplified upon Hsp90 binding, where client proteins are often threaded through its lumen (*20*, *35*, *36*), allowing each domain to fold independently on either side of the chaperone. Furthermore, domain refolding is more productive under conditions where external force is applied (as is the case when the client is DnaK bound) because interdomain misfolding is reduced (*37*). Hence, the client can fold down a smooth folding landscape to the native state without falling into kinetic traps (Fig. 4G). This result can also explain why Hsp70 is involved during the early stages of protein folding (i.e., cotranslationally on the ribosome), whereby interdomain misfolding is unlikely because the complete polypeptide has not emerged from the ribosome tunnel (*32*, *38*). Fewer Hsp70s are likely needed to resolve misfolded states during cotranslational folding because the emerging polypeptide is tethered to the bulky ribosome, thereby enhancing the entropic pulling forces exerted by Hsp70 upon binding; these mechanisms have also been described in the context of protein translocation (*27*). Hsp90 therefore becomes more essential once the entire protein has been translated, whereby the number of available Hsp70 binding sites is much higher and Hsp90 is required to provide a surface whereby folding can occur productively without competition from Hsp70. In addition, both smFRET and simulation data suggest that Hsp90-mediated folding is energetically less expensive compared to Hsp70-mediated folding, consistent with previous observations (*39*). Indeed, we suggest that the “holdase” function of Hsp70 is somewhat of a misnomer (as it implies stable binding) and is energetically expensive because continual and dynamic Hsp70 exchange is required to maintain the expanded chaperone-bound state.

Second, HtpG promotes escape from DnaK-bound states by facilitating a two- to threefold increase (for some domains) in progressive folding events, which are characterized by incremental FRET increases to stable, near-native states. These incremental FRET changes reflect progressive compaction events, as Fluc adopts multiple folding intermediates following escape from the DnaK-bound state, which appear to be regulated by a combination of (i) ATP binding and hydrolysis by HtpG and (ii) the association and dissociation kinetics of DnaK. The mechanism by which Hsp90 uses ATP hydrolysis to promote client folding has been a longstanding and continuing debate in the field (*36*, *40*, *41*); however, our data (and others) now suggest that it is critical in remodeling the client for folding (*21*, *22*). This is likely mediated by the substantial conformational rearrangements that HtpG undergoes upon ATP hydrolysis (*16*), whereby twisting of each monomer can remodel client binding sites in the Hsp90 lumen (*42*, *43*) and/or produce a substantial force on the client such that it can slide through the lumen (*21*). Movement of the client through the lumen, albeit slight, potentially remodels the folding landscape of the protein by promoting local conformational compaction events that prevent misfolding. Optical-tweezer experiments have demonstrated that HtpG induces local chain compaction from an expanded client state following force release (which is mimicked in our system by stochastic DnaK dissociation) in an ATP-hydrolysis–dependent manner (*22*), supporting our conclusion that HtpG mediates progressive folding upon ATP hydrolysis. Recent cryo–electron microscopy data with eukaryotic Hsp90 suggest that both client binding and ATP hydrolysis promote Hsp70 dissociation (*20*, *44*), providing a mechanism by which Hsp90 synchronizes its force-driven folding activity with DnaK release, allowing the client to fold in a progressive manner with access to previously shielded regions. This is likely assisted by the asymmetric nature of ATP hydrolysis on HtpG (i.e., hydrolysis can occur on either subunit) (*20*, *45*, *46*), whereby stepwise remodeling and release of the client promotes folding. While ATP binding and hydrolysis appear to be crucial factors, these experiments cannot exclude the possibility that the ADP-bound form of HtpG is also important in Fluc remodeling. Last, HtpG has been suggested to provide a more hydrophilic surface (compared to DnaK) for folding to occur, which might contribute to the stepwise or gradual compaction of the client observed in this work (*47*).

We were also able to show that progressive folding of the client is highly dependent on DnaK association and dissociation rates. This is largely consistent with a suite of ensemble-based assays, whereby high concentrations of Hsp70 are inhibitory but can be recovered by either reducing the concentration of Hsp70 (*12*), inhibiting Hsp70 (e.g., with telaprevir; this work) or increasing NEF-mediated Hsp70 dissociation (*11*). The reduced rate of DnaK rebinding (mediated by HtpG or telaprevir) increases opportunities for NEF-mediated DnaK dissociation events to be kinetically synchronized, thus allowing the formation of intradomain contacts that would otherwise not occur if bound DnaK was present. This also has the effect of producing smaller conformational rearrangements from the expanded state, facilitating the formation of a folding nucleus from which additional folding events can occur productively with reduced risk of misfolding. An interesting possibility is that the folding intermediates observed during progressive folding represent a transient DnaK-client co-bound state, which partially protects against misfolding and requires subsequent DnaK dissociation to obtain the final native state; however, more work is needed to confirm this. Regardless, while the folding of individual clients via asynchronous DnaK release has been proposed previously (*10*, *48*–*50*), here we have been able to temporally observe it for single client molecules. Critically, we observe progressive folding events under conditions in which Fluc does not typically fold to the native state (e.g. without HtpG), but why does this occur and what is its physiological relevance? Two main possibilities exist; (i) Progressive folding reflects the inherent propensity of the client to fold down an ideal folding landscape to the native state, and (ii) stochastic DnaK dissociation can still produce synchronized folding events as described above, but less frequently. Ultimately, however, progressive folding events are extremely rare under nonoptimal conditions and are generally less productive, providing further evidence that chaperone-mediated folding is required to smoothen folding landscapes.

Our experimental data are also consistent with the kinetic simulations, whereby decreasing DnaK association rates and/or increasing DnaK dissociation rates shifts the binding equilibrium to lower-bound states, thereby increasing opportunities for multiple dissociation events and compaction events to occur. In this instance, optimal folding likely occurs at the transition point between DnaK-mediated conformational expansion (to resolve misfolded states) and compaction of the client (to promote folding). HtpG enhances this process by reducing DnaK association to the folding client, either by promoting more native-like states (with less exposed hydrophobicity) via ATP-hydrolysis–mediated progressive folding or by sterically occluding DnaK from rebinding. Because inhibition of DnaK rebinding is lost when HtpG cannot interact with either DnaK or the client, this suggests that the formation of a DnaK-HtpG-client ternary complex is essential for this mechanism. It is also possible that DnaK-mediated folding (via client association and dissociation) can occur while the client is bound to ATP-bound HtpG, especially because ATP hydrolysis is rate limiting for the HtpG conformational cycle (*51*); however, further work is needed to validate this.

We also note that, while our temporal resolution (∼1 s) is sufficient to capture the dominant Fluc conformational dynamics associated with DnaK binding and folding attempts, faster events cannot be directly resolved in these experiments, necessitating the indirect kinetic analyses performed here. Future higher time-resolution approaches may be valuable for resolving these rapid transitions in greater detail and refining the proposed mechanistic framework. Another important consideration is whether DnaK-mediated inhibition of refolding occurs in vivo, where the pool of free DnaK is likely reduced due to engagement with multiple client proteins. Consistent with this, HtpG is not essential in *E. coli* (*52*), indicating that clients can escape DnaK-mediated inhibition through alternative pathways; however, our data suggest that this would occur less efficiently than when HtpG is present, resulting in slower refolding and increased accumulation of misfolded species. This is further supported by the increased reliance on HtpG during heat stress (*53*), where DnaK concentrations can reach ∼50 μM (*54*) and the burden of misfolded proteins is elevated. In this context, HtpG provides a complementary mechanism to promote client release from DnaK and support productive folding when DnaK alone is insufficient.

Collectively, this work demonstrates that Hsp90 provides a mechanism by which clients can escape the Hsp70-mediated folding block without compromising the ability of Hsp70 to prevent misfolding, in a process primarily driven by ATP hydrolysis. For HtpG, ATP binding alone is sufficient to drive the conformational rearrangements that prime it for ATP hydrolysis (*14*, *51*). However, in eukaryotes, the conformational rearrangements and kinetics of ATP hydrolysis are exquisitely regulated by a complex host of cochaperones (*55*, *56*), which allows the timing and outcome of Hsp90 activity to be fine-tuned according to the diverse folding requirements of individual clients. Despite this additional complexity, our findings indicate that ATP hydrolysis of Hsp90 remains the core mechanism underlying client remodeling, and hence, it is expected that the mechanistic insights provided here are evolutionarily conserved.

## METHODS

### Materials

Plasmids encoding N-terminally SUMO-tagged Fluc mutants with C-terminal AviTag were generated previously (*11*) or modified via site-directed mutagenesis by GenScript. Plasmids encoding chaperones were kindly donated by M. Mayer (University of Heidelberg, Germany).

### Protein expression and purification

Fluc variants were expressed and purified as previously described (*11*) with the following modification: Upon initial purification of the cell lysate via immobilized metal affinity chromatography (IMAC), bound protein was first washed with 10 mM d-biotin for 1 hour to dissociate nonspecific complexes formed between Fluc and biotin-ligase (BirA) before elution with imidazole. DnaK, DnaJ, and GrpE were also purified as described previously (*11*) without modification.

Unless otherwise specified, a modified form of HtpG was used that contains the coiled-coil motif of the kinesin neck region of *Drosophila melanogaster* to the C terminus of HtpG (to maintain a dimeric state) and a C-terminal Strep-Tag II for purification (*13*). Briefly, BL21(DE3) cells containing the plasmid encoding HtpG were grown to mid-log phase (0.6 to 0.8 optical density at 600 nm) in 2× YT medium [tryptone (16 g/liter), yeast (10 g/liter), and NaCl (5 g/liter; pH 7.0)] supplemented with ampicillin (100 μg/ml) at 37°C and protein expression induced by addition of 0.1% (w/v) l-arabinose. Induced cultures were then returned to the orbital shaker for agitation (180 rpm) at 30°C overnight. Cells were subsequently harvested by centrifugation at 5000*g* for 20 min at 4°C, with the obtained cell pellets stored at −20°C until further purification took place. Cells were resuspended in lysis buffer [100 mM tris-HCl (pH 8.0), 150 mM NaCl, 1 mM EDTA, and 2 mM β-mercaptoethanol (BME)] supplemented with cOmplete Protease Inhibitor Cocktail (Sigma-Aldrich) and lysozyme (0.5 mg/ml), and resuspended pellets were left rocking at 4°C for 30 min. Deoxyribonuclease I was added to lysates at a final concentration of 5 mg/ml and incubated at 4°C for a further 30 min with rocking. The lysate was probe sonicated for 3 min (10-s on/20-s off) at 45% power and then clarified via two rounds of centrifugation at 24,000*g* at 4°C for 20 min. The soluble fraction was taken and filtered through a 0.45 μm and loaded onto a 5-ml StrepTrap XT Sepharose column (Cytiva) preequilibrated in lysis buffer. Unbound proteins were then washed from the column with lysis buffer and recombinant protein eluted following the addition of lysis buffer supplemented with 50 mM biotin. Fractions containing recombinant protein were pooled and dialyzed at 4°C overnight into storage buffer [100 mM tris-HCl (pH 7.5), 150 mM NaCl, and 1 mM EDTA]. Dialyzed protein was then concentrated using a Pierce Protein Concentrator (10K MWCO) at 4000*g* at 4°C, snap frozen, and stored at −80°C until required.

HtpG^WT^ (which does not contain the coiled-coil motif), HtpG^E34A^ (which has reduced ATP hydrolysis), HtpG^R355L^ (DnaK-binding defective), and HtpG^W467R^ (impaired interactions with client protein) were expressed in ∆*HtpG* cells (containing a deletion of wild-type HtpG) as described above, with the exception that cells were induced upon addition of 1 mM isopropyl-β-d-thiogalactopyranoside. Recombinant proteins containing the coiled-coil motif and Strep-Tag II (HtpG^E34A^, HtpG^R355L^, and HtpG^W467R^) were purified as described above. For HtpG^WT^, cells were resuspended in IMAC lysis buffer [40 mM Hepes-KOH (pH 7.5), 100 mM KCl, 5 mM MgCl_2_, 10% (v/v) glycerol, 4 mM BME, and 20 mM imidazole] supplemented with cOmplete Protease Inhibitor Cocktail (Sigma-Aldrich) and lysozyme (0.5 mg/ml) and soluble protein extracted as described above. The entire lysate (containing recombinant protein with N-terminal 6xHis-SUMO tag) was loaded onto a 5-ml HisTrap Sephadex column (Cytiva) preequilibrated in HtpG IMAC lysis buffer and washed in the same buffer. Bound recombinant protein was eluted with the addition of IMAC lysis buffer supplemented with 250 mM imidazole. Collected eluate was analyzed via SDS–polyacrylamide gel electrophoresis (PAGE), and protein-containing fractions were pooled and dialyzed at 4°C overnight into cleavage buffer [40 mM Hepes-KOH (pH 7.5), 50 mM KCl, 5 mM MgCl_2_, 10% (v/v) glycerol, 4 mM BME, and 20 mM imidazole] with the addition of ubiquitin-like-specific protease 1 (Ulp1) protease (final concentration: 4 μg/mg of recombinant protein) to remove the 6xHis-SUMO tag. Cleaved protein was then returned to the IMAC column and purified as described above, with fractions containing cleaved recombinant protein pooled and dialyzed into HtpG storage buffer [40 mM Hepes-KOH (pH 7.5), 150 mM KCl, 5 mM MgCl_2_, 10% (v/v) glycerol, and 4 mM BME]. Protein was then concentrated using a Pierce Protein Concentrator (10K MWCO) at 4000*g* at 4°C, snap frozen, and stored at −80°C until required.

### Protein labeling

Fluc constructs were fluorescently labeled with an Alexa Fluor 555 (AF555) and Alexa Fluor 647 (AF647) FRET pair as described previously (*57*) with minor modifications. Briefly, Fluc (2 mg/ml) was incubated in the presence of 5 mM tris(2-carboxyethyl)phosphine and 40% (w/v) ammonium sulfate and placed on a rotator for 1 hour at 4°C. Proteins were then centrifuged at ×20,000*g* for 15 min and resuspended in degassed buffer A [100 mM Na_2_PO_4_ (pH 7.4), 200 mM NaCl, 1 mM EDTA, and 40% (w/v) ammonium sulfate]. The protein was then centrifuged at ×20000*g* for 15 min and resuspended in degassed buffer B [100 mM Na_2_PO_4_ (pH 7.4), 200 mM NaCl, and 1 mM EDTA]. Fluc was then incubated in the presence of a four- and sixfold excess of premixed AF555 donor and AF647 acceptor fluorophores, respectively, and placed on a rotator overnight at 4°C. Following the coupling reaction, excess dye was removed by at least two passes over separate gel filtration columns using a 7000-Da molecular weight cutoff Zeba Spin Desalting column (Thermo Fisher Scientific, USA) equilibrated in 50 mM tris (pH 7.5) supplemented with 20% (v/v) glycerol.

### Refolding assays

The ability of denatured Fluc to spontaneously refold to a native state was assessed by measuring the return of enzymatic activity after denaturation. Denatured Fluc was prepared by incubation of native Fluc in unfolding buffer [50 mM Hepes-KOH (pH 7.5), 50 mM KCl, 5 mM MgCl_2_, 2 mM dithiothreitol (DTT), and 5 M GdHCl] for 30 min at room temperature. Spontaneous refolding of denatured Fluc was initiated by a 1:100 dilution into refolding buffer [50 mM Hepes-KOH (pH 7.5), 80 mM KCl, 5 mM MgCl_2_, 2 mM DTT, and 0.05% (v/v) Tween 20] such that the final concentration of Fluc was 10 nM. Refolding reactions with bacterial chaperones (i.e., KJEG system) were left to incubate at 25°C for up to 120 min. Throughout the refolding reactions, aliquots were taken at various times and dispensed into the bottom of a white 96-well Costar plate (Sigma-Aldrich, USA). The luminescence reaction was initiated following injection of a 10-fold excess of assay buffer [25 mM glycylglycine (pH 7.4), 0.25 mM luciferin, 100 mM KCl, 15 mM MgCl_2_, and 2 mM ATP] into a single well, and 5 s after injection, the luminescence was measured for 10 s using a POLARstar Omega (BMG Labtech, Germany) plate reader. The injection and measurement procedure described above was then performed sequentially for each well to ensure consistency between the measurements. All measurements were performed at 25°C with the gain set at 4000. Refolding yields were calculated by normalizing to the activity of native (nondenatured) Fluc, which was treated as described above with the exception that GdHCl was omitted from the unfolding buffer. Chaperone-assisted refolding reactions were performed as described above with the exception that denatured Fluc was diluted into refolding buffer containing chaperones and nucleotide. Briefly, refolding assays containing chaperones were performed with Fluc in the presence of 0.6 μM DnaJ, 20 μM DnaK, 1.2 μM GrpE (when present), and 10 μM HtpG (when present) supplemented with 5 mM ATP unless otherwise specified. In some instances, greater than 100% refolding activity was observed and is attributed to a small proportion of misfolded (i.e., nonfunctional) Fluc within the native sample.

### TIRF microscopy

#### Microscopy setup

Samples were imaged using a custom-built TIRF microscopy system constructed using an inverted optical microscope (Nikon ECLIPSE Ti2) that was coupled to an electron-multiplied charge-coupled device (EMCCD) camera (Andor iXon Life 897, Oxford Instruments, UK). The camera was integrated to operate in an objective-type TIRF setup with diode-pumped solid-state lasers (200-mW Sapphire, Coherent, USA; or Stradus 637-140, Vortran Laser Technology, USA) emitting circularly polarized laser radiation of 532-nm continuous wavelength. The laser excitation light was reflected by a dichroic mirror (ZT405/488/532/640, Semrock, USA) and focused on the back focal plane of an oil-immersion objective lens (CFI Apochromat TIRF Series 60× objective lens; numerical aperture = 1.49) to create a collimated beam through the sample. Total internal reflection was achieved by directing the incident ray onto the sample at the critical angle (θ_c_) of ∼67° for a glass/water interface. The evanescent light field generated selectively excites the surface-immobilized fluorophores, with the fluorescence emission collected by the same objective lens and filtered by the same dichroic mirror. The emission was then split using a T635lpxr-UF2 dichroic (Chroma, USA), passed through ET690/50m and ET595/50m (Chroma, USA) cleanup filters, and the final fluorescent image projected onto the EMCCD camera. The camera was running in frame transfer mode at 5 Hz, with an electron multiplication gain of 700, operating at −70°C with a pixel distance of 160 nm (in sample space).

#### Coverslip preparation and flow cell assembly

Coverslips were passivated as previously described (*58*), with minor modifications to functionalize the surface with biotin for protein immobilization. Briefly, 24 by 24 mm glass coverslips were cleaned by alternatively sonicating in 100% ethanol and 5 M KOH for a total of 2 hours before aminosilanization in 2% (v/v) 3-aminopropyl trimethoxysilane (Alfa Aesar, USA) for 15 min.

*N*-hydroxysuccinimide–ester methoxy-polyethylene glycol, molecular weight 5 kDa (mPEG) and biotinylated-mPEG (bPEG; Laysan Bio, USA), at a 20:1 (w/w) ratio, were dissolved in 50 mM 4-morpholinepropanesulfonic acid (pH = 7.4) buffer and sandwiched between two activated coverslips for a minimum of 4 hours for initial passivation in a custom-made humidity chamber. PEGylated coverslips were then rinsed with Milli-Q and PEGylated again as described above for overnight (∼20 hours) passivation. PEGylated coverslips were rinsed with Milli-Q water, dried under nitrogen gas, and stored at −20°C until required. Before use, neutravidin (0.2 mg/ml; BioLabs, USA) in Milli-Q was incubated on the passivated coverslip for 10 min to bind to the bPEG. Neutravidin-functionalized coverslips were then rinsed with Milli-Q, dried under nitrogen gas, and adhered to a polydimethylsiloxane flow cell for use in single-molecule experiments. Last, to reduce the nonspecific binding of proteins to the coverslip surface, each channel in the microfluidic setup was incubated in the presence of 2% (v/v) Tween 20 for 30 min as previously described (*59*) and then washed with imaging buffer (refolding buffer supplemented with 6 mM 6-hydroxy-2,5,7,8-tetramethylchroman-2-carboxylic acid).

#### Surface immobilization of labeled proteins and acquisition of smFRET data

For smFRET experiments, labeled Fluc constructs were specifically immobilized to a neutravidin-functionalized and Tween 20–coated coverslip. To do so, labeled proteins (∼ 50 pM final concentration) were diluted in imaging buffer and incubated in the flow cell for 5 min. These conditions would typically give rise to ∼200 to 300 FRET-competent molecules per 100 μm^2^. Unbound proteins were then removed from the flow cell by flowing through imaging buffer.

All data were acquired using the TIRF microscope setup previously described following sample illumination using a 532-nm solid-state laser with excitation intensity of 2.6 W/cm^2^, and the fluorescence of donor and acceptor fluorophores was measured every 200 ms at multiple fields of view. An oxygen scavenging system consisting of 5 mM protocatechuic acid and 50 nM protocatechuate-3,4-dioxygenase was included in all buffers before and during image acquisition to minimize photobleaching and fluorophore blinking.

### smFRET workflow

To benchmark the Fluc sensors for refolding experiments, we measured the FRET distributions of native and misfolded Fluc, the latter generated by removing 4 M guanidine hydrochloride from immobilized Fluc using imaging buffer. Next, Fluc was incubated with DnaJ (0.6 μM) and DnaK (20 μM) for 10 min to generate fully saturated Fluc-DnaK complexes that act as a starting point for each refolding experiment. Image acquisition was initiated, the indicated combination of molecular chaperones was introduced into the microfluidic channel after 10 s (1 ml/min, 5-s dead-time), and the donor and acceptor fluorescence was measured at 10 fields of view for 6 min each (60 min of total imaging time) and used to calculate the FRET efficiency. Unless otherwise specified, the concentrations of each molecular chaperone were as follows: DnaJ (0.6 μM), DnaK (20 μM), GrpE (1.2 μM), and HtpG and variants (10 μM). For experiments in which the DnaK-inhibitor telaprevir (100 μM) or HtpG-inhibitor radicicol (60 μM) were present, the inhibitors were preincubated with the molecular chaperone combinations for 5 min before the addition of ATP to ensure effective binding. In each instance, the concentration of dimethyl sulfoxide was <5%, which did not affect refolding kinetics. All experiments were performed in imaging buffer at room temperature. Where indicated, reactions were supplemented with an ATP regeneration system [5 mM phosphoenolpyruvate and pyruvate kinase (20 ng/μl)] to maintain ATP at steady-state levels throughout the measurement.

### smFRET analysis

#### Molecule selection and FRET calculations

Single-molecule time trajectories were analyzed in MATLAB using the MASH-FRET user interface (version 1.2.2, accessible at https://rna-fretools.github.io/MASH-FRET) (*60*). The apparent FRET value is measured as the ratio between the acceptor fluorescence intensity (*I*_Acceptor_) and the sum of both donor (*I*_Donor_) and acceptor fluorescence intensities after correcting for cross-talk between donor and acceptor channels. The formula for calculating the apparent FRET efficiency is given by Eq. 1, whereby the corrected acceptor intensity (denoted as *CI*_Acceptor_) is equal to *I*_Acceptor_ – (*g* × *I*_Donor_) and *g* is the cross-talk correction constant. *g* is calculated as the ratio of fluorescence measured in the acceptor and donor detection channels following direct excitation of a protein labeled with a single donor fluorophore. There was negligible nonspecific excitation of the acceptor observed with the 532-nm laser

$$\text{FRET efficiency}=\frac{{\mathrm{CI}}_{\text{Acceptor}}}{{\mathrm{CI}}_{\text{Acceptor}}+I_{\text{Donor}}}$$ (1)

Briefly, donor and acceptor fluorescence channels were aligned following a local weighted mean transformation of images of TetraSpeck fluorescence beads and donor and acceptor fluorescence spots colocalized to identify FRET pairs. Molecules that displayed clear donor and/or acceptor photobleaching events or demonstrated anticorrelated changes in donor and acceptor fluorescence intensity were selected for subsequent analysis. The number of photobleaching events observed was used to determine the number of fluorophores present; only molecules in which a single donor and acceptor photobleaching event was observed were used for further analysis. While κ^2^ = ^2^/_3_ is generally considered a reasonable assumption for dynamic averaging, we did not experimentally determine dye orientation in this system; therefore, reported distances should be interpreted relatively rather than absolutely.

#### Trace processing and HMM fitting

Selected molecules were denoised in MASH-FRET using the nonlinear filter, which has been described previously (*61*), to accurately identify and quantify transitions between different FRET states during downstream processing. Parameter values were as follows: exponent factor for predictor weight: 5, running average window size: 1, and factor for predictor average window sizes: 5. Data were truncated to only include FRET values acquired before donor or acceptor photobleaching. FRET efficiency data were exported to the state finding algorithm vbFRET (version vbFRET_nov12; https://sourceforge.net/projects/vbFRET/) and trajectories fit to an HMM to identify discrete FRET states, their residence times, and the transition distributions between them. Owing to the complexity of the system and the many conformations that Fluc can adopt during chaperone-assisted folding, the maximum number of states for HMM fitting was set to 5. Default vbFRET settings were used to fit data to the HMM, with the exception that the mu and beta hyperparameters were changed to 1.5 and 0.5, respectively, to prevent overfitting.

#### Kinetic analysis of HMM fits

The HMM fits of individual FRET trajectories were further analyzed to extract key kinetic information arising from changes in Fluc conformation. To investigate transitions of interest, each transition (as determined from the HMM analysis) was sorted into different directional classes denoted generally as *T*_Before-After_, whereby “before” refers to *F*_Before_ and “after” refers to *F*_After_. For simplicity, FRET data were binned according to whether *F*_Before_ or *F*_After_ was >0.3 (high) or <0.3 (low), unless otherwise indicated, and thus, two different transition classes are possible: *T*_high-low_ and *T*_low-high_. The residence time (defined as the time that a molecule resides at *F*_Before_ before transition to *F*_After_) for each transition within a transition class was calculated and presented as cumulative histograms or the mean ± SEM. Because the FRET data could be well described as a two-state system in the context of chaperone binding (i.e., bound or nonbound), the cumulative residence times (determined as the period of time that the HMM fit resides at low-FRET states before a *T*_low-high_ transition, and vice versa for *T*_high-low_ transitions) are presented. The *F*_After_ following transitions away from DnaK-bound states (upon either DnaK dissociation, client folding, or a combination of both) was then determined from the HMM fits and shown as a violin plots or cumulative distributions. The resultant distributions were then tested for statistical differences using a Kolmogorov-Smirnov approach with bootstrapping (250 data points per condition, repeated 200 times), with statistical difference determined if the median *P* value was <0.05. Last, we identified transitions away from DnaK-bound states (i.e., when the FRET increased from <0.3 to >0.3) that had *T*_low-high_ of at least 10 s. These events were classified as progressive if the subsequent transition was a further increase in FRET (i.e., an increase in FRET after it had already increased above 0.3) or nonprogressive (i.e., if FRET remained unchanged until photobleaching or if the next transition was a decrease).

Because it is not possible to determine for how long a particular FRET state would have existed if not truncated because of photobleaching, the last measured FRET state was deleted and thus excluded from residence time calculations. Because data were smoothed during denoising, residence times shorter than that given by Eq. 2 were not considered for further analysis. Frames represent the imaging frame rate in milliseconds, and *N*_FA_ is the number of frames that were averaged during trace denoising

$${\text{Residence time}}_{\text{Delete}}=2\cdot \text{Frames}\cdot N_{\text{FA}}$$ (2)

The residence times were fit with a double-exponential model and the two rates (*k*_1_ and *k*_2_) and the contribution of each rate to the final fit determined. The accuracy of the fitting was validated using a bootstrapping approach, where residence times were sampled, fitted to a double exponential, and the mean and SE of *k*_1_ and *k*_2_ were determined by taking the mean distribution of fit values from each bootstrap attempt (*n* = 500). Last, to determine whether there are changes in FRET immediately before or after chaperone dissociation, all *T*_low-high_ transitions were identified (which are indicative of chaperone dissociation), filtered such that only transitions with a residence time > 10 s were selected, and the FRET efficiency 10 s before and after each *T*_low-high_ was plotted. The FRET efficiency of all data points for each molecule was collated and presented as FRET efficiency kernel density estimate distributions or heatmaps.

#### Histogram fitting to determine proportion of native, misfolded, or DnaK-bound states

To determine the proportion of time that Fluc resided in native, misfolded, or DnaK-bound states, data from the FRET efficiency histograms were fit to a mixed Gaussian model as previously described (*11*). Briefly, native or misfolded states of Fluc were fit to Gaussians without parameter constraints in the absence of chaperones, and the resultant sigma, center, and gamma (for the skewed Gaussian model) parameter values were used to constrain subsequent mixed Gaussian fits of the smFRET data acquired in the presence of chaperones. The area under each curve was determined and normalized to the combined area of all Gaussians curves to determine the proportion of each Fluc state.

### Modeling of smFRET data

To determine whether experimentally observed smFRET data could be explained by a model in which DnaK dynamically transitions between different DnaK-bound states, theoretical simulations were performed. The observed DnaK-association rate was estimated by fitting the decrease in FRET efficiency following a nonprogressive folding event (for Fluc^IDS1^ in the absence of HtpG, i.e., KJE; described above) to a single exponential, yielding a rate constant of *k*_on_ = 0.55 s^−1^. Because it was hypothesized that *k*_1_ from double-exponential fits of *T*_low-high_ residence times more accurately reflects DnaK-dissociation times (see above), the average *k*_1_ across all Fluc sensors was used to approximate the DnaK dissociation rate (*k*_off_ = 0.15 s^−1^).

Using these *k*_on_ and *k*_off_ values, we simulated transitions between different DnaK-bound states (denoted as state*_n_*, where *n* is the number of bound DnaK proteins) every 1 s over a total of 360 s, consistent with the timescales of smFRET acquisitions. On the basis of the entropic-pulling model of Hsp70 function (*27*, *62*), we assumed that at least two or more DnaK proteins are required for full conformational expansion of the client. Accordingly, FRET states were assigned as follows: low-FRET when ≥2 DnaK proteins were bound and high-FRET when one or none were bound. *T*_low-high_ residence times were then extracted from >500 simulated FRET trajectories as previously described for smFRET data, and the resulting distributions were plotted as cumulative density plots and fit using either single- or double-exponential models. To assess whether the number of potential DnaK-binding sites influences the simulated residence times, simulations were repeated with increasing maximum numbers of DnaK-bound states (state_max_). The average *T*_low-high_ residence time and the proportion of time spent in each state*_n_* were also determined as a function of state_max_*.*

### Binding site prediction

The Fluc sequence (P08659) was screened for potential DnaK-binding residues using three established prediction tools, ChaperISM (*30*), Paladin (*29*), and the LIMBO web server (*31*), with threshold values of 3, −9, and the “best overall prediction mode,” respectively, used to convert each tool’s output to a per-residue binary annotation (1 = predicted binder, 0 = not). The binary annotation was parsed by custom Python scripts to identify predicted binding regions. To do this, consecutive binder residues were grouped and retained as a binding patch only if the number of consecutive predicted binding residues was > 2, and the patch was discontinued only when the intervening nonbinding residues were > 4.

### Statistical analysis

When comparing the means between multiple conditions, data were statistically analyzed using a one-way analysis of variance (ANOVA) with a Tukey’s multiple-comparison post hoc test, with *P* ≤ 0.05 determined to be statistically significant. All data analysis and presentation were performed using custom-written scripts on the Python software or using GraphPad Prism 9 (GraphPad Software Inc., San Diego, USA).
