# Supplementary material for: Direct single-molecule visualization of Hsp90-mediated relief of an Hsp70-folding block
Source: Sci Adv. 2026 Jul 29;12(31):eaeg5464. doi: 10.1126/sciadv.aeg5464 (PMC13418734; doi:10.1126/sciadv.aeg5464)
Supplement: Supplementary file 1 — Figs. S1 to S8 [file sciadv.aeg5464_sm.pdf]

Science Advances
AAAS

Supplementary Materials for

Direct single-molecule visualization of Hsp90-mediated relief of an Hsp70-folding block

Nicholas R. Marzano *et al.*

Corresponding author: Nicholas R. Marzano, nick.marzano@chem.ox.ac.uk; Heath Ecroyd, heathe@uow.edu.au

Sci. Adv. **12**, eaeg5464 (2026)
DOI: 10.1126/sciadv.aeg5464

This PDF file includes:

Figs. S1 to S8

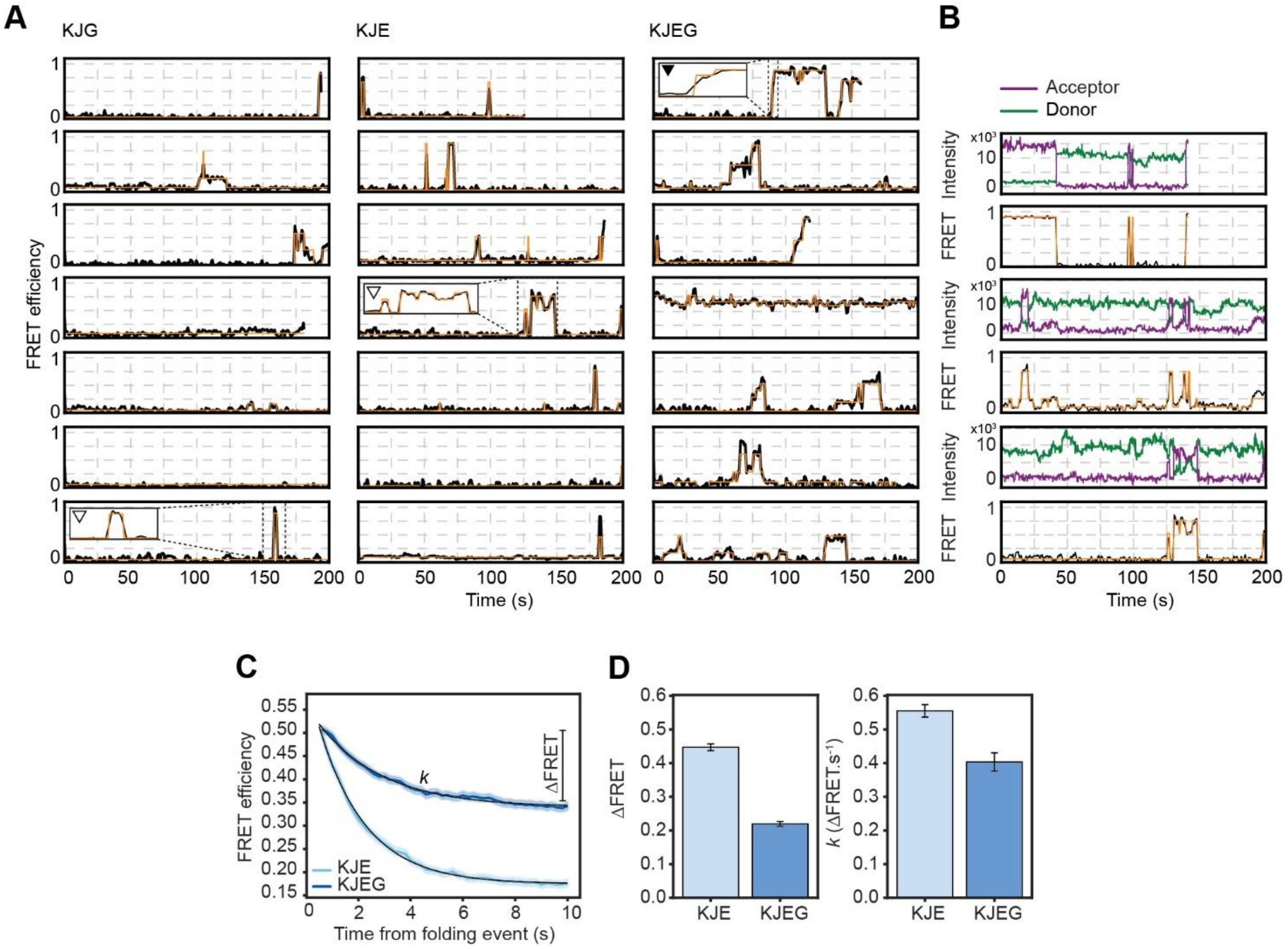

Fig S1: HtpG reduces the rate of DnaK-rebinding and promotes efficient client refolding following escape from the DnaK-bound state. (A) Example FRET trajectories from individual Fluc^{IDS1} molecules upon incubation with the indicated combination of molecular chaperones (HMM shown in orange). Insets show a zoomed visualization of either progressive (*filled triangle*) or non-progressive transitions (*empty triangle*). **(B)** Raw fluorescence and FRET trajectories from Fluc molecules with FRET spikes, without denoising. Clear fluorescence anti-correlation demonstrates that the observed events are due to changes in FRET. **(C)** Exponential fit of the FRET efficiency immediately following non-progressive events from Fig 1G. **(D)** Both the change in FRET (*left*) and rate constant *k* (*right*) determined from the exponential fits in panel C are shown. Data are presented as mean ± standard deviation of fit.

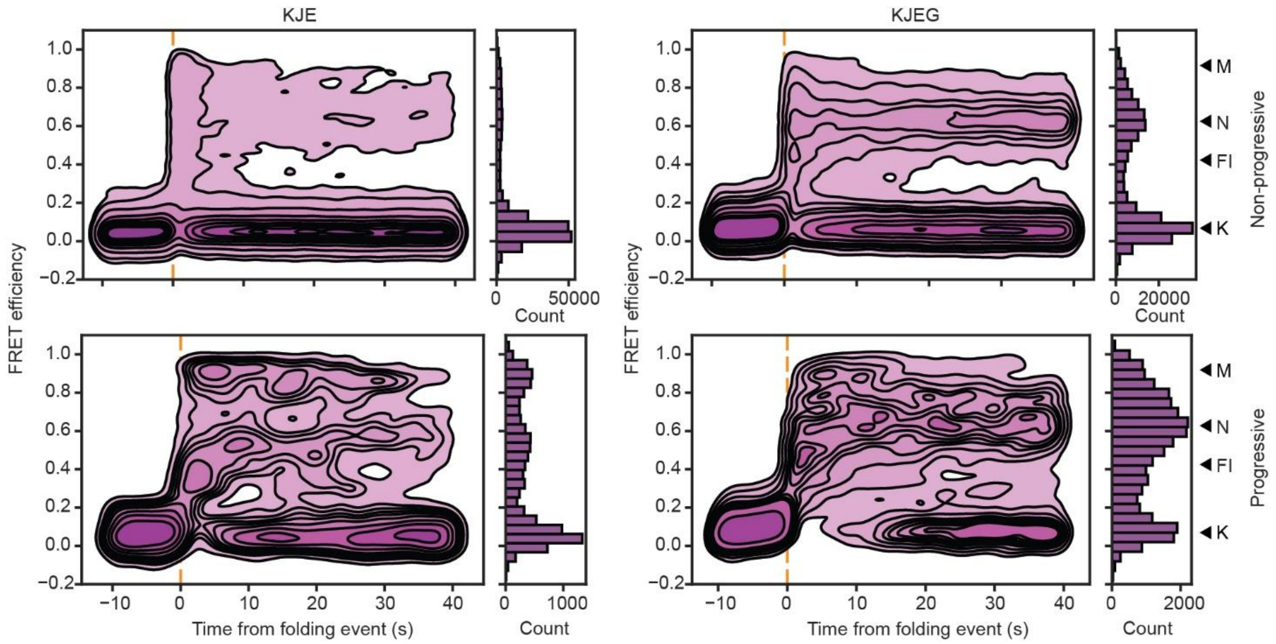

Fig S2: HtpG-mediated progressive folding of Fluc^{IDS1} results in the client forming an intermediate state to prevent misfolding and favor folding towards the native state. 2D FRET efficiency heatmaps following non-progressive (*top*) or progressive (*bottom*) folding events from the DnaK-bound state in the absence (KJE, *left*) or presence of HtpG (KJEG, *right*). Histograms show the collated FRET efficiency following the folding events (i.e., > 0 s). Keys to the right indicate the FRET value corresponding to misfolded (M), native (N), folding intermediate (FI) or DnaK-bound (K) Fluc^{IDS1} states.

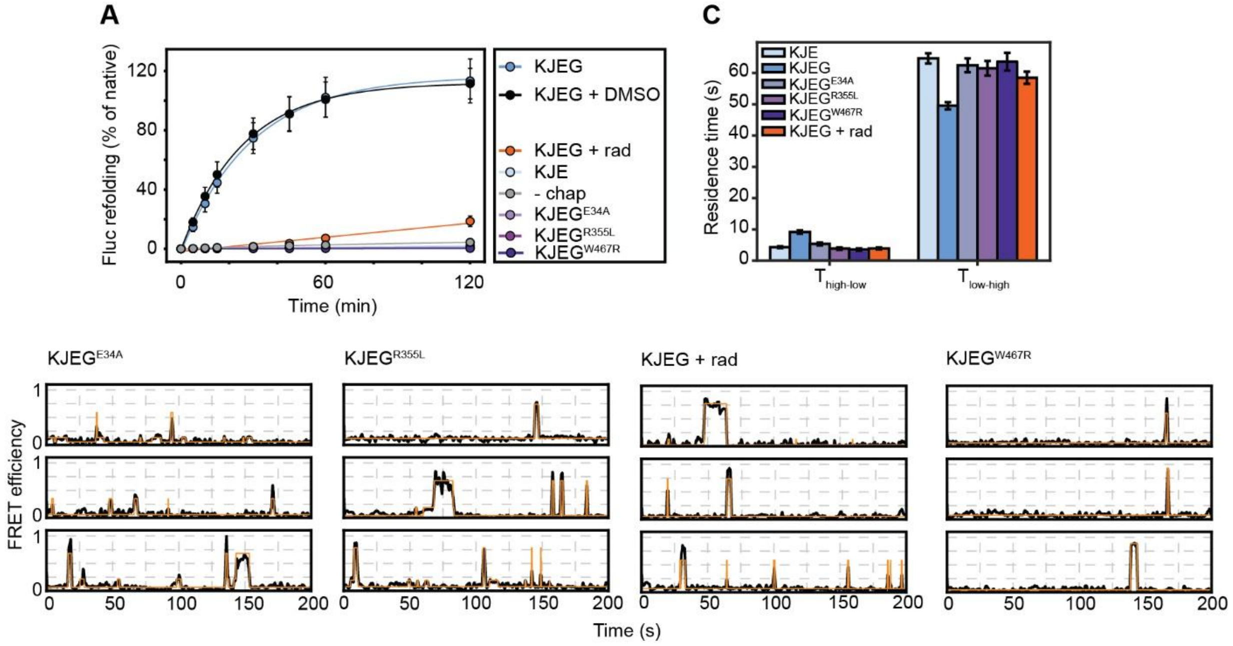

Fig S3: HtpG interacts with DnaK and hydrolyzes ATP during productive client refolding. (A) Luciferase refolding assay in the absence or presence of the KJEG system containing the indicated HtpG mutants or the HtpG inhibitor, radicicol (60 µM). Fluc^{IDS1} was diluted 100-fold into refolding buffer alone (i.e., no chap) or supplemented with various combinations of molecular chaperones. All treatments contained 5 mM ATP. Data shown represents the mean ± SEM from three independent experiments. **(B)** Example FRET trajectories from individual Fluc^{IDS1} molecules upon incubation with the indicated combination of molecular chaperones (HMM shown in orange). **(C)** Residence time data showing the time Fluc^{IDS1} remains in a DnaK-bound state (i.e., < 0.3 FRET) prior to a non-bound state (i.e., $T_{low-high}$) and vice-versa. Data are presented as mean ± SEM from at least three independent experiments.

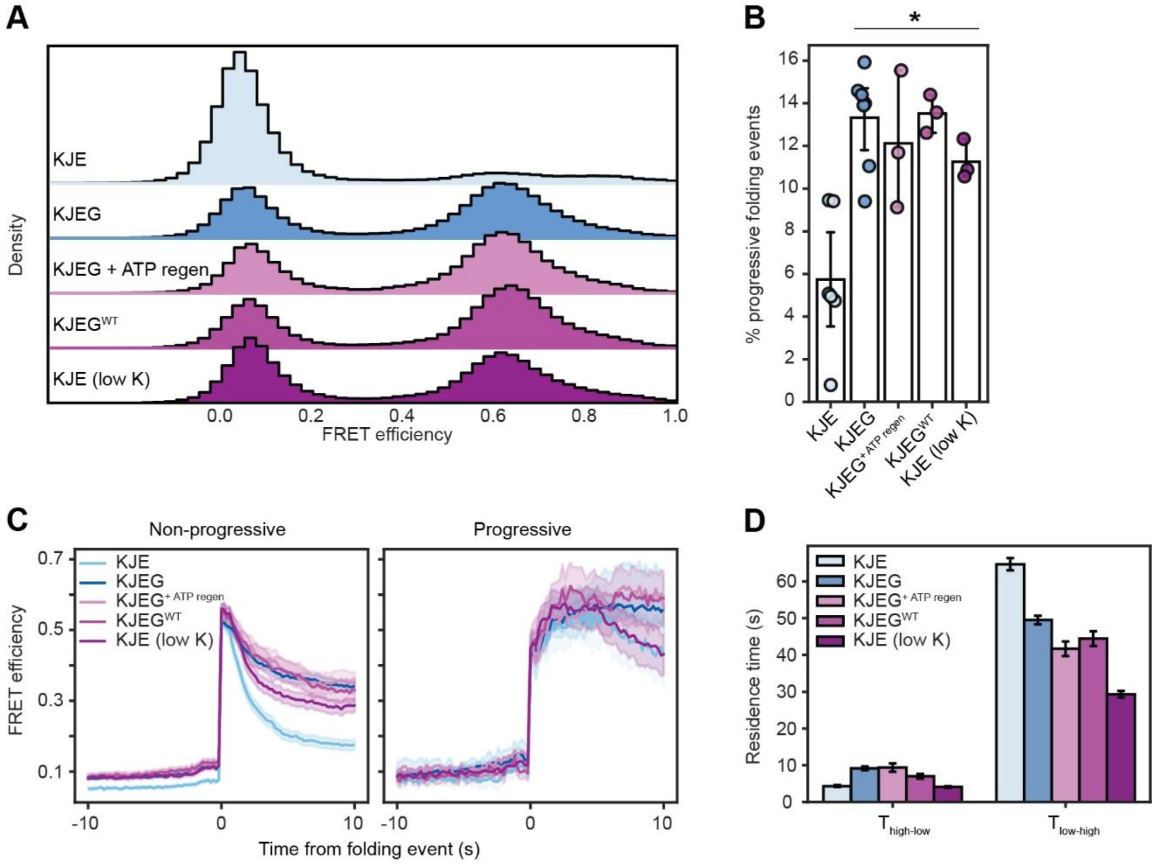

Fig S4: Coiled-coil HtpG exhibits similar ability to refold Fluc^{IDS1} compared to non-modified HtpG^{WT} or when supplemented with an ATP regeneration system. Fluc^{IDS1} was refolded in the presence of DnaJ (0.6 µM), DnaK (20 µM), GrpE (1.2 µM) alone (*KJE*) or supplemented with coiled-coil HtpG or HtpG^{WT} (10 µM) with 5 mM ATP (*KJEG* or *KJEG^{WT}*, respectively). Notably, HtpG^{WT} does not contain the StrepTagII motif, while coiled-coil HtpG does. To determine whether ADP accumulation could influence the observed results, KJEG-mediated refolding was performed in the absence or presence of an ATP regeneration system (*KJEG + ATP regen*). Lastly, to validate that the KJE system is capable of Fluc^{IDS1} refolding under optimized conditions, refolding in the presence of the KJE system was performed with a lower concentration of DnaK (5 µM, *KJE [low K]*). **(A)** FRET efficiency histograms in the presence of the indicated combinations of chaperones. Data are compiled from at least 456 individual molecules per condition. We note that the presence of the coiled-coil and StrepTagII motif on HtpG does not affect its refolding ability at the single-molecule level compared to HtpG^{WT}. **(B)** The percentage of progressive folding events, shown as the mean ± SEM. A one-way ANOVA with Tukey's post-hoc test was performed, with * denoting statistical significance of $p < 0.05$. **(C)** The average FRET efficiency prior to and after non-progressive (*left*) and progressive (*right*) folding events ± SEM. Data are compiled from at least 373 events per condition. **(D)** Residence time data showing the time Fluc^{IDS1} remains in a DnaK-bound state (i.e., < 0.3 FRET) prior to a non-bound state (i.e., $T_{low\text{-}high}$) and *vice-versa*, shown as the mean ± SEM.

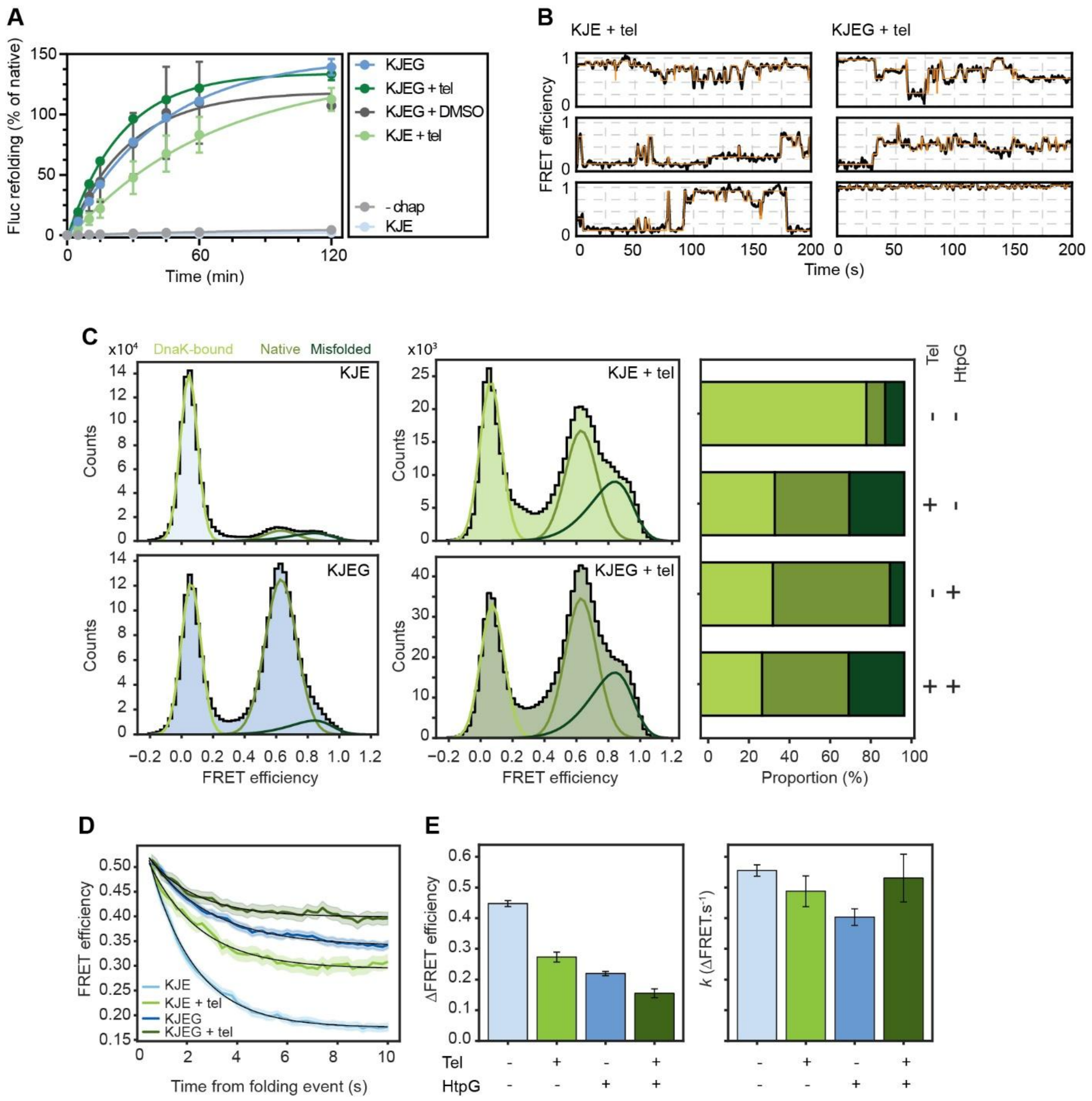

Fig S5: HtpG mimics the telaprevir-induced inhibition of DnaK to enable client folding but does not prevent DnaK rebinding to misfolded proteins. (A) Luciferase refolding assay in the absence or presence of the KJE or KJEG system that has been previously incubated with or without the DnaK-inhibitor telaprevir (100 µM). Fluc[IDS1] was diluted 100-fold into refolding buffer alone (i.e., no chap) or supplemented with various combinations of molecular chaperones. All treatments contained 5 mM ATP. Data shown represents the mean ± SEM from three independent experiments. **(B)** Example FRET trajectories from individual Fluc[IDS1] molecules upon incubation with the indicated combination of molecular chaperones and the DnaK-inhibitor telaprevir (100 µM, HMM shown in orange). **(C)** FRET efficiency histograms with the multiple-Gaussian model fits used to determine the proportion of Fluc[IDS1] states (i.e., native, misfolded or DnaK-bound, *left*) during chaperone-assisted refolding. The proportion of each state is shown (*right*) in the presence of the KJE system with or without HtpG and/or telaprevir. **(D)** Exponential fit of FRET efficiency immediately following non-progressive DnaK-release from Fig 2H. **(E)** Both the change in FRET (*left*) and rate constant *k* (*right*) as determined from the exponential fits in panel C are shown. Data for Fluc[IDS1] in the absence or presence of HtpG (i.e., KJE or KJEG) without telaprevir is the same as presented in Fig 1.

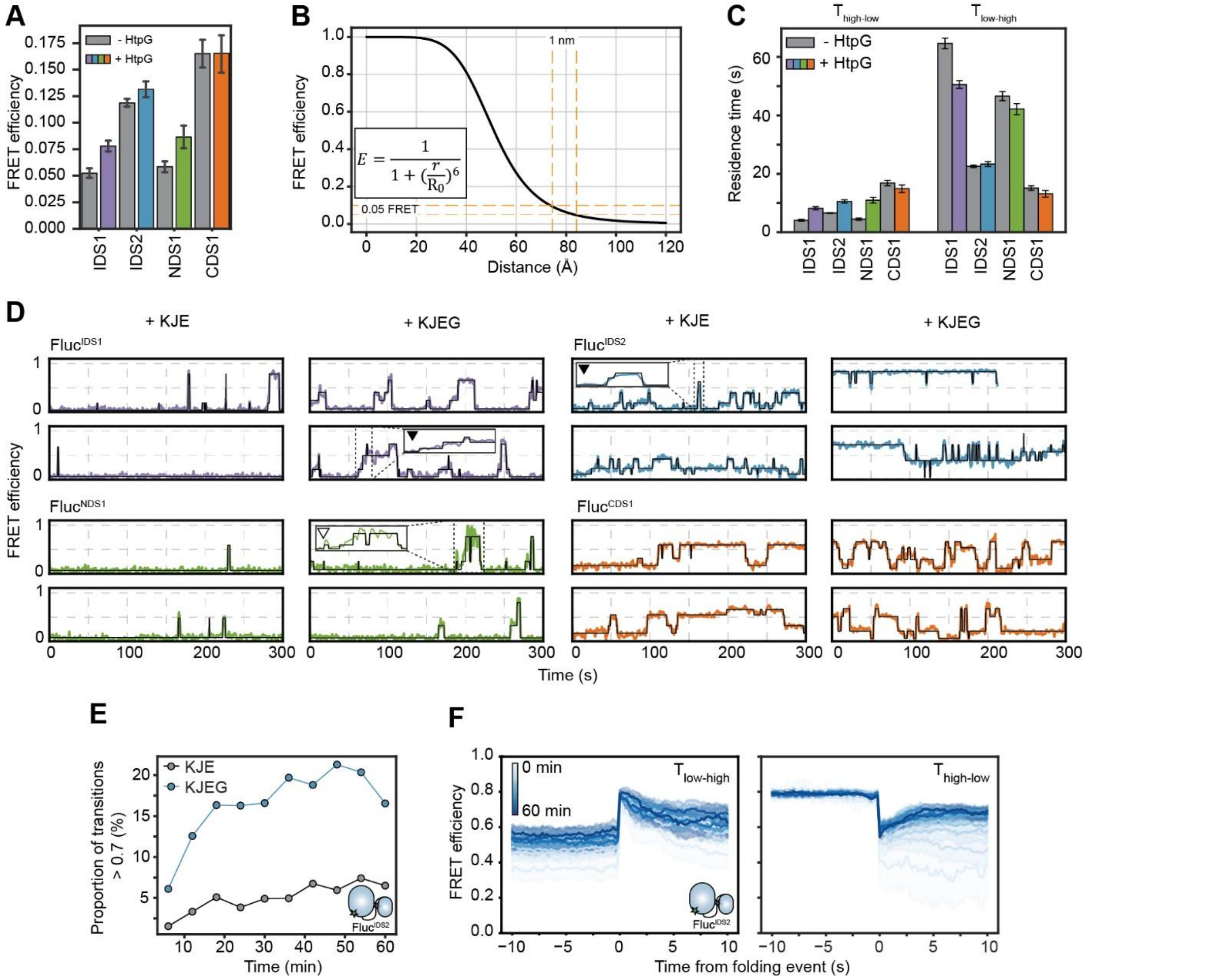

Fig S6: Kinetics of HtpG-mediated folding of multiple Fluc domains. (A) FRET efficiency of each Fluc sensor in the absence or presence of HtpG prior to folding events. **(B)** Non-linear dependence of FRET efficiency with distance. The FRET curve assumes a Forster radius of 51 Å (equivalent to that of an AF555/AF647 FRET-pair). Dotted lines demonstrate that the small change in FRET observed in panel A reports on a large difference in distance. The FRET equation is also shown, where *r* and R_0 are the distance between fluorophores and the Forster radius, respectively. **(C)** Residence time data showing the time each Fluc sensor remains in a DnaK-bound state (i.e., < 0.3 FRET) prior to a non-bound state (i.e., $T_{low-high}$) and vice-versa in the absence or presence of HtpG. Data are presented as mean ± SEM. **(D)** FRET trajectories for each Fluc sensor when incubated in the presence of the KJE system only or when supplemented with HtpG. Insets show a zoomed visualization of either progressive (*filled triangle*) or non-progressive transitions (*empty triangle*). **(E)** The proportion of transitions to the native $Fluc^{IDS2}$ state (> 0.7 FRET) in the absence or presence of HtpG over time. **(F)** The average FRET efficiency of all transitions towards (*left,* $T_{low-high}$) or away from (*right,* $T_{high-low}$) the native $Fluc^{IDS2}$ state (> 0.7 FRET) during refolding.

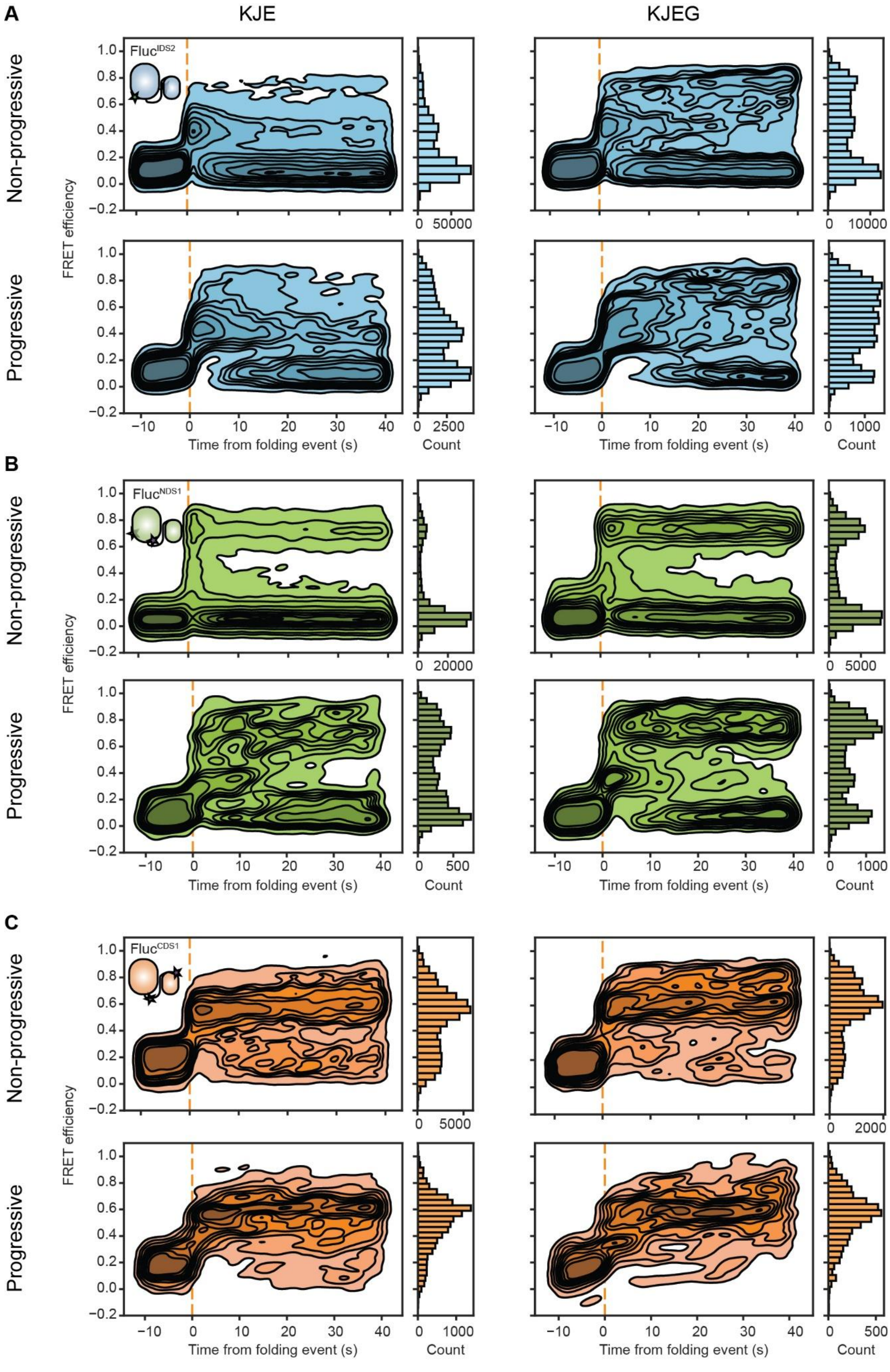

Fig S7: HtpG-mediated progressive folding guides multiple regions of the client through productive intermediates and prevents misfolding. 2D FRET efficiency heatmaps following non-progressive (*top*) or progressive (*bottom*) folding events following transition from the ultra-low FRET DnaK-bound state in the absence (KJE, *left*) or presence of HtpG (KJEG, *right*) for **(A)** $Fluc^{IDS2}$, **(B)** $Fluc^{NDS1}$ and **(C)** $Fluc^{CDS1.}$ Histograms show the collated FRET efficiency following the folding events (i.e., > 0 s).

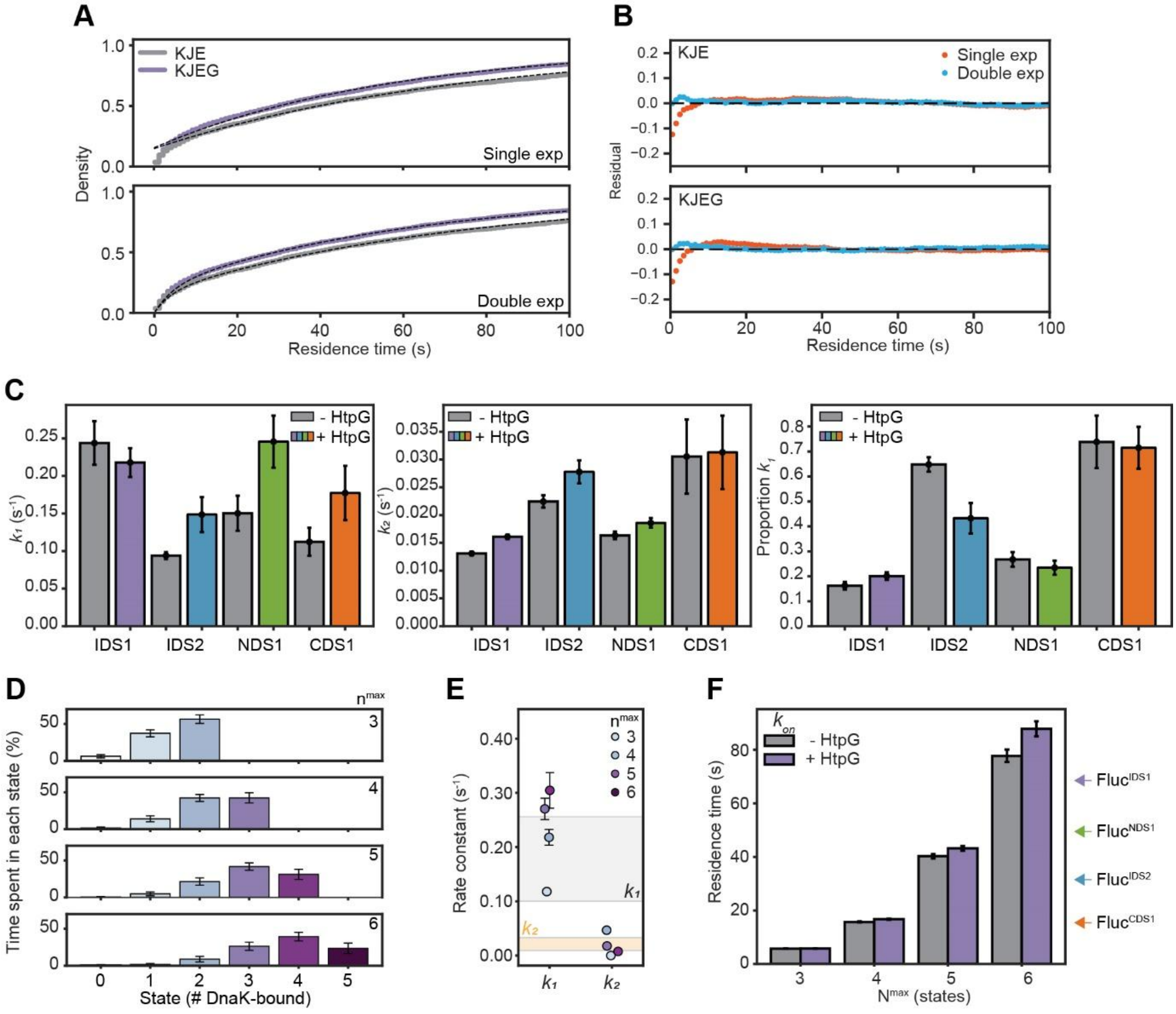

Fig S8: Conformational compaction of Fluc upon DnaK-release is described by two rate constants. (A) Cumulative density of $Fluc^{IDS1}$ $T_{low\text{-}high}$ residence times (from Fig S3D) in the absence or presence of HtpG, fit to either a (*top*) single- or (*bottom*) double-exponential model. **(B)** The residuals for both the single- and double-exponential fits from panel A are shown. **(C)** The extracted rate constants from the double-exponential fits for all Fluc sensors in the absence or presence of HtpG, with k_1 (*left*) and k_2 (*middle*) denoted. The proportion of the total fit that is described by k_1 is also shown (*right*). **(D)** The percentage of time that Fluc resides in each DnaK-bound state as a function of $state_{max}$, based on > 500 trace simulations. Data represents the mean ± standard deviation. **(E)** Predicted k_1 and k_2 rate constants following a double-exponential fit of T_{bound} residence times from simulated FRET trajectories at different $state_{max}$. Experimental rates for k_1 and k_2 determined from smFRET experiments are shown for comparison. Data represents the mean ± SEM. **(F)** The DnaK association rate (i.e., k_{on}) was determined in the absence or presence of HtpG (from Fig S1B-C) and used to simulate transitions between different DnaK-bound states and theoretical FRET trajectories. Simulations were performed with increasing $state_{max}$ and the mean $T_{low\text{-}high}$ residence times ± SEM from simulated FRET trajectories are shown. Mean $T_{low\text{-}high}$ residence times from smFRET experiments for each Fluc sensor in the absence of HtpG are shown on the right for comparison.
